# EEF2K silencing inhibits tumour progression through repressing SPP1 and synergises with BET inhibitors in melanoma

**DOI:** 10.1002/ctm2.722

**Published:** 2022-02-20

**Authors:** Guangtong Deng, Furong Zeng, Yi He, Yu Meng, Huiyan Sun, Juan Su, Shuang Zhao, Yan Cheng, Xiang Chen, Mingzhu Yin

**Affiliations:** ^1^ Department of Dermatology Hunan Engineering Research Center of Skin Health and Disease Hunan Key Laboratory of Skin Cancer and Psoriasis Xiangya Hospital Central South University Changsha Hunan China; ^2^ National Clinical Research Center for Geriatric Disorders Xiangya Hospital Central South University Changsha Hunan China; ^3^ Department of Oncology Xiangya Hospital Central South University Changsha Hunan China; ^4^ Department of Pharmacy The Second Xiangya Hospital Central South University Changsha Hunan China

**Keywords:** BET inhibitors, EEF2K, melanoma, SPP1, STAT3

## Abstract

**Background:**

Despite the remarkable breakthroughs achieved in the management of metastatic melanoma using immunotherapy and targeted therapies, long‐term clinical efficacy is often compromised due to dose‐limiting toxicity and innate or acquired resistance. Therefore, it is of vital importance to further explore the molecular mechanisms underlying melanoma progression and identify new targeted therapeutic approaches.

**Methods:**

The function of eukaryotic elongation factor‐2 kinase (EEF2K) in melanoma were investigated in vitro and in vivo. RNA‐seq and chromatin immunoprecipitation (ChIP) assay were undertaken to explore the mechanisms. The antitumor effect of bromodomain and extra terminal domain (BET) inhibitors combined with cytarabine were assessed in melanoma both in vitro and in vivo.

**Results:**

EEF2K silencing markedly attenuated the malignant phenotypes of melanoma cells, including proliferation, migration, invasion and metastasis. In contrast, EEF2K overexpression promoted melanoma cell proliferation, migration and invasion. Mechanistically, we demonstrated that EEF2K upregulates the phosphorylation of STAT3 (p‐STAT3) at Tyr705, which binds to the promoter region of SPP1 and enhances its transcription, thus facilitating melanoma progression. Transfection‐induced re‐expression of SPP1 partly negated the inhibitory effect of EEF2K silencing on melanoma, whereas inhibition of SPP1 or STAT3 significantly abolished the efficacy of EEF2K on melanoma cells. Intriguingly, EEF2K silencing combined with BET inhibitor treatment further inhibited cell proliferation and promoted apoptosis in melanoma. We further screened the US FDA‐approved antitumour drug library and identified cytarabine as a potential clinically applicable EEF2K inhibitor that could synergise with BET inhibitors in melanoma treatment.

**Conclusion:**

EEF2K/p‐STAT3/SPP1 may be a novel oncogenic pathway in melanoma progression, which could be a target for novel combination therapy for melanoma.

## Graphical abstract

Graphical Highlights:

1. EEF2K silencing attenuates melanoma progression.

2. EEF2K mediates melanoma progression through p‐STAT3/SPP1 axis.

3. Cytarabine, a potential clinically applicable EEF2K inhibitor, synergises with BET inhibitors in melanoma treatment.

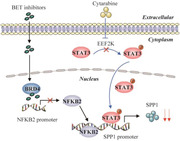

## 1 INTRODUCTION

Melanoma is the most aggressive malignant tumour among cutaneous malignancies.^1^ Although current intensive approaches, such as targeted therapy and immunotherapy, have shown great promise in the treatment of metastatic or unresectable melanoma, patient outcomes remain dismal due to dose‐limiting toxicity and innate or acquired resistance.^2^ Therefore, it is crucial to further explore the underlying mechanism of melanoma progression and to identify new targeted therapeutic approaches, as well as deployment of a combination therapy to achieve a superior sustainable antitumour response.

Eukaryotic elongation factor‐2 kinase (EEF2K), a calcium/calmodulin‐dependent protein kinase, acts as a negative regulator of protein synthesis by suppressing the elongation stage via a phosphorylation pathway to inactivate the eukaryotic translation elongation factor 2.^3^ Numerous studies have demonstrated that EEF2K is involved in various tumour‐associated processes, including cell cycle, apoptosis, angiogenesis, epithelial–mesenchymal transition and sensitivity to chemotherapies in tumour progression.^4^, ^5^ The close association between EEF2K and tumour progression highlighted EEF2K as an attractive target for cancer therapy. Interestingly, EEF2K plays a paradoxical role in tumour progression. For example, EEF2K plays a supportive role in several cancers, such as breast cancer,^6^ glioblastoma,^7^ oesophageal squamous cell carcinoma^8^ and ovarian cancer,^9^ but a suppressive role in colorectal and lung cancers.^10^, ^11^ Moreover, EEF2K was shown to be involved in multiple processes independent of its role as a regulator of protein synthesis,^11^ highlighting the importance of investigating the underlying mechanisms for its diverse regulatory roles. Considering that the role of EEF2K in melanoma has never been elucidated, it is imperative to clarify the regulatory function of EEF2K in melanoma progression.

Secreted phosphoprotein 1 (SPP1) is an integrin‐binding phosphorylated glycoprotein that has been involved in different biological functions, including cell adhesion, migration and invasion.^12^, ^13^, ^14^ We previously showed that SPP1 was highly expressed in melanoma, and SPP1 silencing inhibited melanoma cell proliferation, migration and invasion. We further demonstrated that bromodomain and extra‐terminal domain (BET) inhibitors impeded melanoma progression through an SPP1‐dependent pathway.^15^ However, the upstream regulatory mechanism of SPP1 and whether it enhances the inhibitory effects of BET inhibitors on melanoma remain largely elusive.

In this study, we found that EEF2K might play a role in the regulation of melanoma progression through the p‐STAT3/SPP1 pathway. Additionally, we screened the US FDA‐approved antitumour drugs library and identified cytarabine as a potential clinically applicable EEF2K inhibitor that has a synergistic effect with BET inhibitors on melanoma treatment. Our findings revealed that EEF2K/p‐STAT3/SPP1 might be a novel oncogenic pathway in melanoma progression and could serve as an important reference point for potential combination therapy for melanoma.

## 2 METHODS

### 2.1 Cell culture

SK‐MEL‐28, A375, A2058, WM35 and HEK293T cells were purchased from American Type Culture Collection (ATCC, Manassas, VA, USA) and cultured in Dulbecco's Modified Eagle's Medium (DMEM, Biological Industries, Kibbutz Beit‐Haemek, Israel) supplemented with 10% fetal bovine serum (Biological Industries, Kibbutz Beit‐Haemek, Israel) and 1% penicillin‐streptomycin solution (Beyotime Biotechnology, Shanghai, China). All cultured cells were incubated at 37°C in humid air with 5% CO_2_.

### 2.2 RNA interference

siRNAs and negative controls were purchased from GenePharma (Shanghai, China) or Ruibobio (Guangzhou, China). In brief, cells were seeded in six‐well culture plates at 70% confluence and transfected with target siRNAs using Turbofect based on the manufacturer's instructions. Cells were harvested and verified by real‐time PCR and Western blotting. The target sequences are shown as follows: EEF2K: GCTCGAACCAGAATGTCAA; STAT3: GCAACAGATTGCCTGCATT; CAACATGTCATTTGCTGAA.

### 2.3 Quantitative real‐time PCR

Total RNA was extracted using TRIpure reagent (BioTek, VT, USA) and then reverse transcribed using HiScript Q RT SuperMix kit (Vazyme, Nanjing, China). Real‐time PCR was performed with SYBR Green Master Mix (CWBIO, Jiangsu, China) in Applied Biosystems QuantStudio 3 Real‐Time PCR System (Thermo Fisher Scientific, MA, USA). Gene expressions were normalised to GAPDH expression. Primers are summarised in Table S1.

### 2.4 Western blotting and co‐immunoprecipitation

To determine the interactions between endogenous EEF2K and STAT3, 10 million cells were harvested using NP‐40 buffer (Beyotime Biotechnology, Shanghai, China) and incubated with 4 μL primary antibody against each partner protein, respectively, along with 40 μL protein A/G beads (Beyotime Biotechnology, Shanghai, China), and the immunoprecipitates were eluted with 2× SDS loading buffer. For immunoblotting, total cell lysates were harvested with RIPA lysis buffer (Beyotime Biotechnology, Shanghai, China). The cell lysates were separated by 10% SDS–PAGE and transferred to polyvinylidene fluoride membrane. The membranes were then blocked with 5% skim milk for 1 h at room temperature and incubated with the primary antibody at 4°C overnight, followed by secondary antibodies (ABclonal, MA, USA) for 1 h at room temperature. The bands were visualised with Western ECL Blotting Substrates. Antibodies used were as follows: EEF2K (Cell Signaling Technology, MA, USA), SPP1 (ProMab, CA, USA), STAT3 (Cell Signaling Technology, MA, USA), p‐STAT3 (Tyr705, Ser727) (Cell Signaling Technology, MA, USA), PKM2 (Cell Signaling Technology, MA, USA), IgG (Beyotime Biotechnology, Shanghai, China) and ACTIN (Santa Cruz Biotechnology, TX, USA).

### 2.5 Cell viability assay

Cells were seeded into 96‐well plates at an appropriate density per well. Cell viability was calculated by the Cell Counting Kit‐8 (CCK‐8) assay (Bimake, TX, USA) at different time points. The absorbance values were detected by microplate reader (BioTek, VT, USA) at a wavelength of 450 nm. The combination index (CI) was calculated using the CompuSyn software based on the Chou–Talalay methodology, and less than 1 indicates synergy.^16^

### 2.6 Cell cycle and cell apoptosis

Cell cycle was performed by flow cytometry using cell cycle kit (Beyotime Biotechnology, Shanghai, China). Briefly, cells were fixed with 70% ethanol and stained with propidium iodide. Cell distribution was assessed by ModFit LT software. Cell apoptosis was performed with Annexin V‐AF647/PI kit (4A Biotech, Beijing, China) and analysed by flow cytometry.

### 2.7 Wound‐healing assay

Cells were plated into six‐well plates and were scratched straightly with a P‐10 pipette tip to generate a wound when the cells reached 90% confluence. After phosphate‐buffered saline (PBS) washing, the medium was replaced with fetal bovine serum‐free DMEM medium. Images were taken under microscope at indicated time points after scraping. Image J was used to quantify the wound surface area.

### 2.8 Transwell invasion assay

Cells in serum‐free medium were seeded in an 8.0‐μm transwell chamber inserts (Corning, NY, USA) precoated with matrigel (Corning, NY, USA). Medium containing 10% FBS was added at the lower chamber. After 24‐h incubation, the inserts were fixed with 4% paraformaldehyde for 15 min and stained with 0.5% crystal violet for 15 min. Cells on the top of the inserts were scraped with a cotton swab. The migrated cells were examined by microscope.

### 2.9 Animal study

All animal experiments were conducted under protocols approved by the Ethical Review of Experimental Animals at Central South University. Pathogen‐free NOD‐SCID‐gamma (NSG) mice (6–8 weeks old) were purchased from the Department of Laboratory Animals, Central South University.

In xenograft model, 10^6^ cells were suspended in 100 μL PBS and then inoculated subcutaneously into the right flank of each NSG mouse. For the therapy experiments, 10^6^ SK‐MEL‐28 cells were injected subcutaneously. Tumour‐bearing mice were randomised in groups and treatments began when the tumour size reached 50–100 mm^3^. Cytarabine (Selleck, Shanghai, China) was prepared in saline and administrated through intraperitoneal injection. NHWD‐870 was dissolved in corn oil and administrated orally. Tumour‐bearing mice were treated with vehicle, cytarabine (30 mg/kg), NHWD (0.75 mg/kg) or a combination of both drugs from day 9 to day 21 using a regimen in which drugs were administered for 2 days, stopped for 1 day and then the process repeated for the duration of the treatment. Tumour size and bodyweight were recorded every 3 days. Tumour size was measured using a digital caliper and calculated as [(length × width × width)/2].

For the lung metastasis model, 10^6^ cells were suspended in 100 μL PBS and injected through caudal vein. Animals were sacrificed around 30 days after inoculation and the lung tissues were collected. Metastasis nodules were examined by haematoxylin–eosin (H&E) staining.

### 2.10 Immunohistochemistry (IHC)

Formalin‐fixed, paraffin‐embedded tumour slides were dewaxed, rehydrated and repaired with boiled citrate buffer. After quenching endogenous peroxide activity with hydrogen peroxide and blocking the nonspecific binding with goat serum, the slides were incubated with primary antibody (anti‐ki67, Abcam, MA, USA; anti‐EEF2K, Abcam, MA, USA) at 4°C overnight in a humidified chamber. After incubation with secondary antibody, the slides were developed with DAB and counterstained with haematoxylin. Images were photographed using microscope and quantified by Image J. IHC evaluation of tissue array was identified by two independent pathologists. EEF2K expression was determined by assessment of staining intensity and percentage of positive cells, as we described previously.^17^

### 2.11 TUNEL assay

Apoptotic tumour cells were quantified by TUNEL assay (Beyotime Biotechnology, Shanghai, China). Briefly, Formalin‐fixed, paraffin‐embedded tumour slides were dewaxed, rehydrated and incubated with DNase‐free proteinase K (20 μg/ml, Beyotime Biotechnology, Shanghai, China) for 30 min at room temperature. After three times wash, the tissues were incubated with TUNEL working solution for 1 h at 37°C and counterstained with DAPI (Servicebio, Wuhan, China). Positively stained cells were examined by microscope.

### 2.12 Lentiviral transduction and RNA‐sequencing

Short hairpin RNA (shRNA) vectors to deplete EEF2K were purchased from GENECHEM (Shanghai, China). EEF2K and SPP1 vector were purchased from Fenghui Biotechnology (Changsha, China). Briefly, the vectors and package plasmid (Addgene, MA, USA) were cotransfected into HEK293T cells using TurboFect (Thermo Fisher Scientific, MA, USA) according to the manufacturer's instructions. After 2 days, the viral‐containing supernatants were used to transduce target cells. Transduced cells were selected in the presence of puromycin (Thermo Fisher Scientific, MA, USA) at 2 μg/mL for 3 days.

Total RNA from EEF2K knockdown and control SK‐MEL‐28 cells was extracted with Trizol (Invitrogen, CA, USA) and used for RNA‐seq analysis. Library construction and data processing were performed by Beijing Genomics Institute. Libraries were sequenced on a BGISEQ‐500RS. Only genes with at least one read in each of four samples and at least 40 reads in total among all samples were retained for following analyses.^18^ Differential expression genes were defined with |log2 fold‐change| > 1 and *Q*‐value <.05 and was used for the ingenuity pathway analysis.

### 2.13 Chromatin immunoprecipitation (ChIP)‐qPCR

SK‐MEL‐28 cells were cross‐linked with 1% formaldehyde and incubated with glycine (0.125 M) to terminate the cross‐linking. Consequently, the cells were collected with lysis buffer and sonicated by a Bioruptor to generate an average chromatin fragment size of 100–750 bp. The sonicated samples were immunoprecipitated with STAT3 (Cell Signaling Technology, MA, USA) or IgG (Beyotime Biotechnology, Shanghai, China) antibodies, followed by washing and reversal of cross‐linking. Purified DNA was examined by real‐time PCR. Primers used were as follows: SPP1 forward: TCCCTGTGTTGGTGGAGGAT, reverse: ACTGAAGCTGTACCTTGGTCG; BCL2L10 (−664/−516) (positive control) forward: CTAAGACAGCTGCCAAGTGC, reverse: TCCATTCTGCATCAGTCTGG; BCL2L10 (−2157/−2025) (negative control) forward: CTTTGGAGGGAGAATTCCA, reverse: CAGATGGACAGAATTACATGC.

### 2.14 In vitro kinase assay

Recombinant GST‐tagged EEF2K, kinase dilution buffer I/III and Ca^2+^/Calmodulin solution II were purchased from SignalChem. Recombinant GST‐tagged STAT3 and ATP stock solution were purchased from Novus Biologicals and Cell Signaling, respectively. To determine the role of EEF2K in the phosphorylation of STAT3, recombinant GST‐tagged STAT3 was incubated with active recombinant GST‐tagged EEF2K according to the manufacturer's protocols. Phosphorylation of STAT3 was detected by p‐STAT3 (Tyr705, Ser727) antibody.

### 2.15 Statistical analyses

All the data were analysed with GraphPad Prism 8 (GraphPad Software, La Jolla, CA, USA). Results are representative of three independent experiments and presented as mean ± standard deviation. Two‐tailed unpaired Student's *t*‐test was employed for comparisons between two groups. ANOVA analysis was performed to calculate *p*‐values between multiple groups. Nonparametric tests were applied if the data were not normally distributed. The detailed statistical methods were indicated in corresponding figures. Kaplan–Meier survival curves were calculated and the log‐rank test was used to determine the differences between groups. A *p*‐value of <.05 was considered to indicate a statistically significant difference.

## 3 RESULTS

### 3.1 EEF2K silencing suppresses melanoma cell proliferation and tumour growth

**FIGURE 1:**
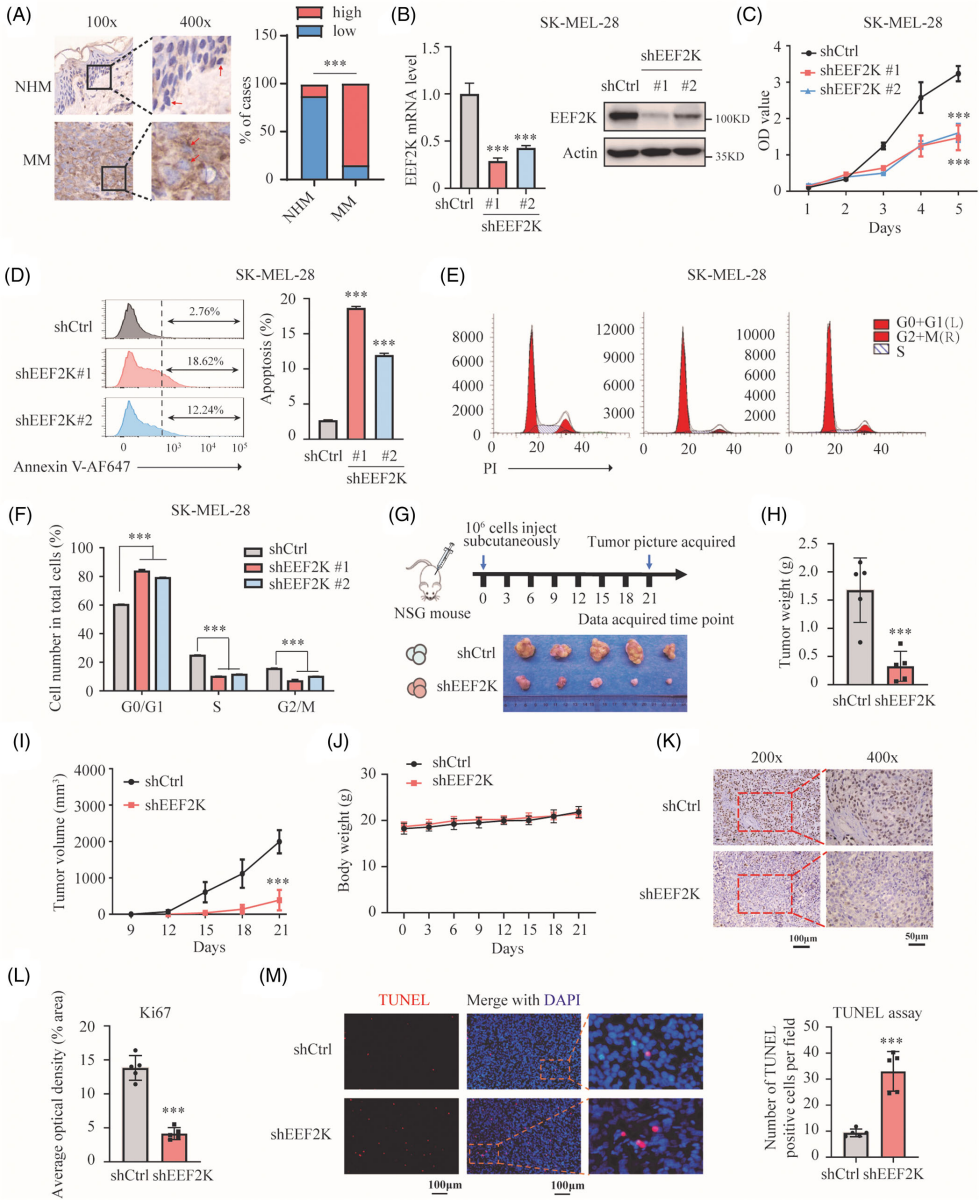
EEF2K silencing suppresses melanoma cell proliferation and tumour growth. (A) EEF2K expression in normal human melanocytes (NHM) and melanoma in tissue array quantified by immunohistochemistry. *N* (normal skin) = 8, NHM are marked with red arrows; *N* (malignant melanoma) = 40, melanoma cells are marked with red arrows. MM, malignant melanoma. (B) EEFK2 knockdown efficiency quantified by real‐time PCR and Western blotting. (C–F) Cell proliferation (C), cell apoptosis (D), and cell cycle distribution (E and F) of SK‐MEL‐28 cells after EEF2K silencing. (G) Schematic view of the xenografted model and picture of the resected xenografted tumours (*n* = 5 in each group). (H–J) Tumour weight (H), tumour volume (I) and bodyweight (J) of mice in the indicated groups. (K and L) Ki67 staining of the sectioned tumours to identify tumour cell proliferation. (M) TUNEL assay to quantify apoptotic cells in xenografted tumours. *p*‐Value was calculated using Fisher's exact test in (A). One‐way ANOVA analysis was performed in (B), (D) and (F). Two‐way ANOVA analysis was performed in (C) and (I). Two‐tailed unpaired Student's *t*‐test was performed in (H) and (L). Unpaired *t*‐test with Welch's correction was performed in (M). ***p < .001

The role of EEF2K has been reported in many types of tumours, but its role in melanoma remains unclear. To investigate the role of EEF2K in melanoma, we first checked its expression using tissue array and found that EEF2K was overexpressed in melanoma (Figure 1A). Then we conducted GSEA according to the EEF2K expression profile in the Cancer Genome Atlas (TCGA)‐SKCM dataset. Results indicated that melanoma‐related pathways were significantly enriched in the high EEF2K group (*p* < .001) (Figure S1A). To further explore the biological functions of EEF2K in melanoma, we performed EEF2K silencing in four melanoma cell lines using shRNA, and cell proliferation was significantly suppressed as detected using the CCK‐8 assay (Figure 1B,C and Figure S1B–F). We further examined the effects of EEF2K silencing on apoptosis and cell cycle. Results showed that EEF2K silencing increased the percentage of apoptotic cells and cell arrest in the G0/G1 phase in both SK‐MEL‐28 and A375 melanoma cells, and decreased the cell percentage in S and G2/M phases (Figure 1D–F and Figure S1G,H). To evaluate whether EEF2K inhibition would be toxic to normal melanocytes, we knockdown EEF2K in PIG1 cells (Figure S1I), finding that cell proliferation and apoptosis were not affected (Figure S1J,K).

To further clarify the role of EEF2K in vivo, EEF2K‐silenced SK‐MEL‐28 cells were injected subcutaneously into the right flank of NSG mice (Figure 1G). We found that EEF2K silencing caused significant tumour growth and volume delay in xenografted melanoma tumours (Figure 1G–I), but the bodyweight was comparable between the control and EEF2K silencing groups (Figure 1J). IHC for the proliferation marker (Ki67) revealed fewer proliferative cells in EEF2K knockdown xenograft tumours compared to that in the control samples (Figure 1K,L). TUNEL assay indicated significantly increased number of apoptotic cells in EEF2K knockdown xenograft tumours (Figure 1M). To investigate the pro‐oncogenic role of EEF2K in melanoma, we overexpressed EEF2K in melanoma cells and confirmed that EEF2K overexpression promotes melanoma cell proliferation in vitro and in vivo (Figure S2A–G). Collectively, our results revealed that EEF2K silencing suppresses melanoma cell proliferation and tumour growth, and overexpression of EEF2K increases cell proliferation and tumour growth.

### 3.2 EEF2K deficiency impairs melanoma cell migration, invasion and metastasis

**FIGURE 2:**
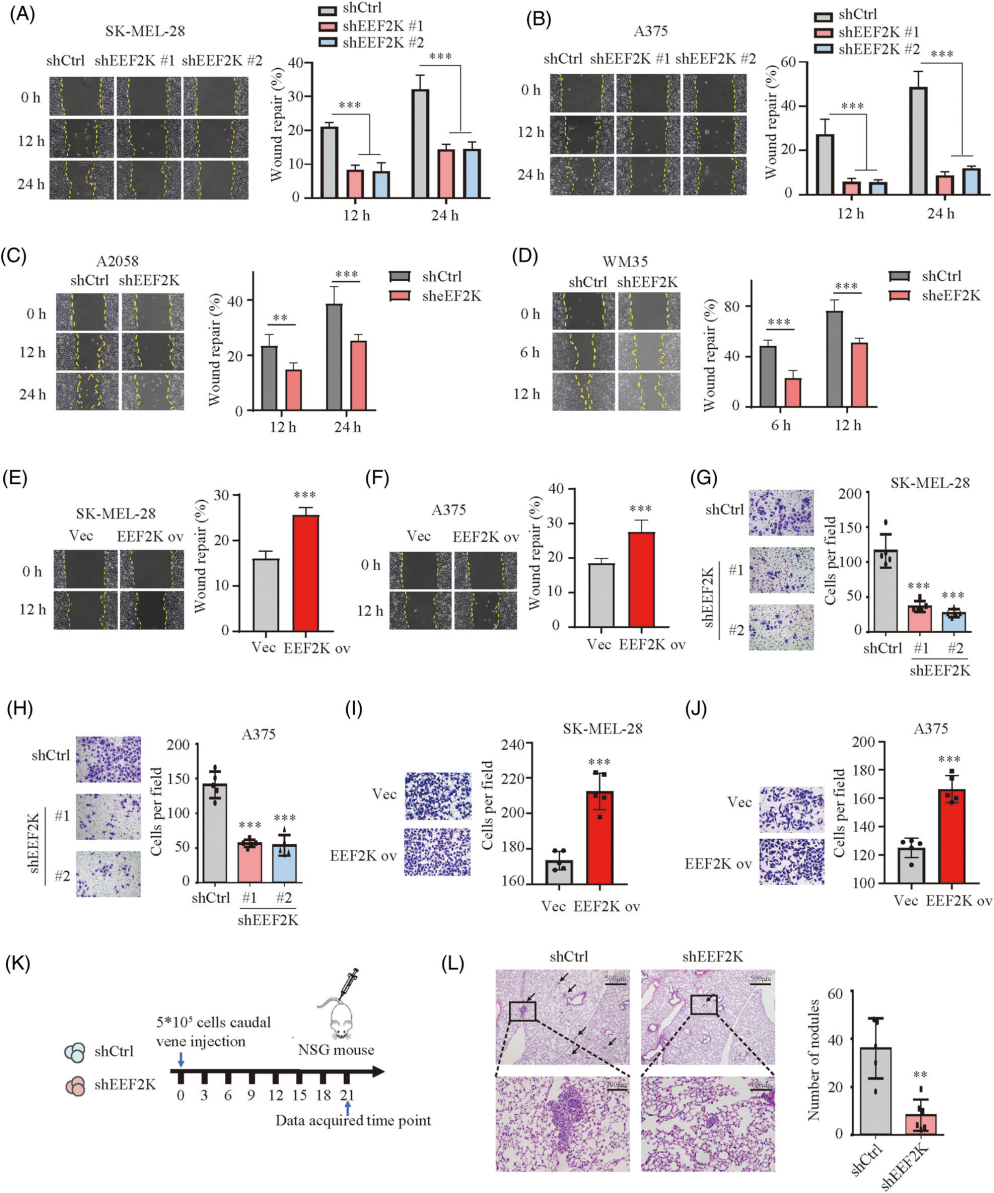
EEF2K deficiency impairs melanoma cell migration, invasion and metastasis. (A–D) Wound healing assay identifying the migratory capacity after EEF2K silencing in SK‐MEL‐28 (A), A375 (B), A2058 (C) and WM35 cells (D). (E and F) Wound healing assay identifying the migratory capacity after EEF2K overexpression in SK‐MEL‐28 (E) or A375 (F) cells. Five random areas were selected. Images (at 100× magnification) were taken at the indicated time points. (G–J) Transwell assay quantifying the invasive capacity after EEF2K silencing in SK‐MEL‐28 (G) or A375 (H) cells, and after EEF2K overexpression in SK‐MEL‐28 (I) or A375 (J) cells. Invaded cells were determined for 18–24 h. Five random areas were selected. Images were taken at 200× magnification. (K) Schematic view of the lung metastasis model. (L) Metastatic nodules in lung indicated by haematoxylin–eosin staining. Two‐way ANOVA analysis was performed in (A)–(D). One‐way ANOVA analysis was performed in (G) and (H). Two‐tailed unpaired Student's *t*‐test was performed in (E), (F), (I), (J) and (L). **p < .01; ***p < .001

Cell motility and migration are important for melanoma progression, invasiveness and metastasis.^19^ We sought to verify whether EEF2K affects melanoma cell migration and invasion by utilising wound healing and transwell assays. Delayed wound repair was detected in EEF2K‐silenced melanoma cells (Figure 2A–D). Conversely, EEF2K overexpression led to a significantly increased migratory capacity (Figure 2E,F). Likewise, the number of invasive melanoma cells was also significantly decreased in EEF2K silencing and increased in EEF2K overexpression group (Figure 2G–J). We further validated our findings using a lung metastasis model, which could mimic the latter half of the metastatic process. SK‐MEL‐28 cells expressing shcontrol or shEEF2K were injected via the caudal vein (Figure 2K). Mice injected with EEF2K deficient melanoma cells showed a decrease in metastatic burden, with fewer lung micro‐metastases per lung section, as detected using H&E staining of the tumour nodule specimens (Figure 2L). Overall, these findings suggested that EEF2K deficiency impairs melanoma cell migration, invasion and metastasis.

### 3.3 EEF2K regulates SPP1 expression and STAT3 signalling pathway

**FIGURE 3:**
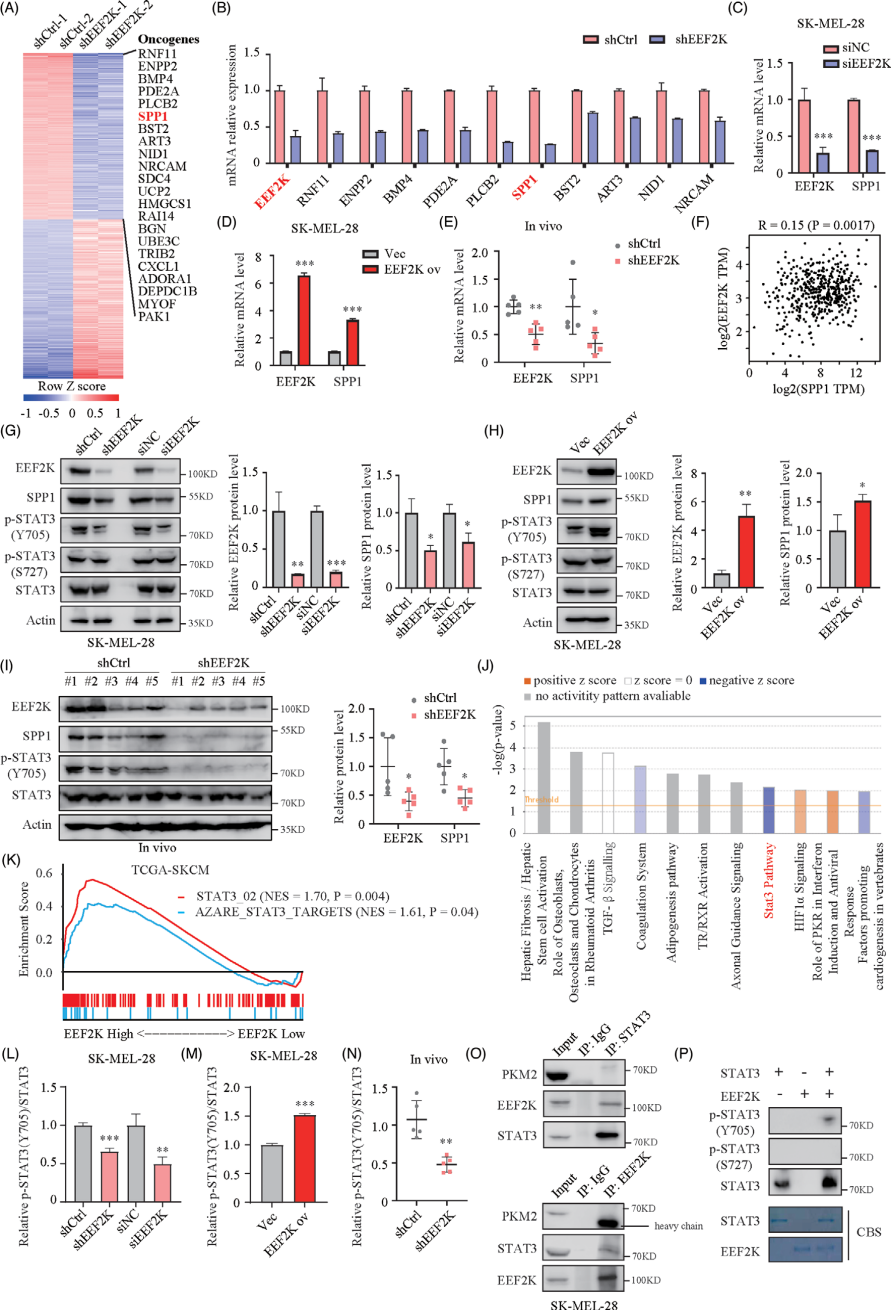
EEF2K regulates SPP1 expression and STAT3 signalling pathway. (A) Heatmap of the differentially expressed genes in EEF2K knockdown compared to control SK‐MEL‐28 cells. The melanoma‐promoting genes reported previously were listed on the right side. (B) Verification by real‐time PCR of the top 10 differentially expressed melanoma‐promoting genes in SK‐MEL‐28 cells. (C and D) SPP1 mRNA expression in SK‐MEL‐28 cells after EEF2K silencing with siRNA (C) or EEF2K overexpression (D). (E) SPP1 mRNA expression in EEF2K knockdown tumour xenografts compared to control group. (F) Correlation analysis between EEF2K and SPP1 expression in TCGA‐SKCM dataset from Gene Expression Profiling Interactive Analysis dataset. (G and L) Quantification by Western blotting of SPP1, p‐STAT3 and STAT3 expression after EEF2K knockdown in SK‐MEL‐28 cells. (H and M) Quantification by Western blotting of SPP1, p‐STAT3 and STAT3 expression after EEF2K overexpression in SK‐MEL‐28 cells. (I and N) Quantification by Western blotting of SPP1, p‐STAT3 and STAT3 expression in EEF2K knockdown tumour xenografts compared to control group. (J) Ingenuity pathway analysis of the RNA‐seq data. (K) Gene set enrichment analysis results of TCGA‐SKCM dataset based on EEF2K expression. (O) Cell lysates of SK‐MEL‐28 cells were immunoprecipitated with EEF2K or STAT3 antibodies against each protein, then immunoblotted with the indicated antibodies. (P) In vitro phosphorylation assay was performed by incubation of recombinant 1 μg EEF2K and 1 μg STAT3 in the corresponding buffer for 1 h. P‐STAT3 was detected by Western blotting. CBS, Coomassie brilliant blue staining. *p*‐Values were calculated using two‐tailed unpaired Student's *t*‐test. Comparison of EEF2K protein level between shCtrl and shEEF2K groups was assessed using unpaired *t*‐test with Welch's correction in (G). *p < .05; **p < .01; ***p < .001

To uncover the underlying target of EEF2K in the regulation of melanoma cell proliferation and metastasis, we performed RNA sequencing in EEF2K knockdown and control SK‐MEL‐28 cells. GSEA indicated that cell cycle, DNA replication and vascular endothelial growth factor signalling pathway were significantly inhibited in the EEF2K silencing group (Figure S3), which is consistent with our previous findings. A heatmap of the differentially expressed genes, including the previously reported melanoma‐promoting genes, was generated (Figure 3A, Table S2). We validated the top 10 differentially expressed melanoma‐promoting genes using real‐time PCR assay, which yielded results consistent with sequencing assay (Figure 3B). Among these genes, SPP1 caught our attention, not only because it was ranked in the top 10 and was downregulated most significantly based on the real‐time PCR results, but also it was identified as a crucial melanoma driver in our previous study.^15^ Consistently, SPP1 mRNA levels were decreased after EEF2K silencing with siRNA and increased after EEF2K overexpression in both A375 and SK‐MEL‐28 cells (Figure 3C,D and Figure S4A,B). In addition, a significant decrease in SPP1 mRNA level was found in EEF2K knockdown tumour xenografts (Figure 3E). Additionally, there is a weak but significantly positive correlation between EEF2K and SPP1 at the mRNA level, based on the TCGA‐SKCM dataset (Figure 3F). Most importantly, these findings were consistent at the protein level, indicating that EEF2K positively regulates SPP1 expression (Figure 3G–I and Figure S4C,D).

To clarify the difference in canonical pathways between the EEF2K knockdown and control groups, we performed ingenuity pathway analysis and found that STAT3 signalling was significantly downregulated in EEF2K‐deficient cells (Figure 3J). Consistently, GSEA results based on the TCGA‐SKCM datasets validated our findings, indicating that the STAT3 signalling pathway and targets were activated in the high‐EEF2K group (Figure 3K). Phosphorylation of STAT3 is known to be important for its dimerisation, translocation and transcriptional activation. Intriguingly, transient or stable knockdown of EEF2K resulted in notable downregulation of p‐STAT3 (Y705), and overexpression of EEF2K led to increased p‐STAT3 (Y705) without affecting total STAT3 and p‐STAT3 (S727) (Figure 3G–I,L–N and Figure S4C,D). EEF2K has been reported to reduce the expression of PKM2 in breast cancer,^20^ and interact with PKM2 to regulate the phosphorylation of STAT3 in lung cancer.^11^ However, the mRNA and protein level of PKM2 were unchanged after EEF2K silencing (Figure S4E). Moreover, co‐immunoprecipitation assay demonstrated that endogenous EEF2K was precipitated with endogenous STAT3, but not with PKM2, suggesting that EEF2K promotes the phosphorylation of STAT3 in a PKM2‐independent manner in melanoma cells (Figure 3O). Considering that EEF2K is a protein kinase and interact with STAT3, to determine whether EEF2K served as a protein kinase for STAT3, we used in vitro kinase assay and found that incubation of STAT3 with active EEF2K led to phosphorylation of STAT3 at Tyr705 but not Ser727 (Figure 3P). These results demonstrated that EEF2K positively regulates the STAT3 signalling pathway through directly phosphorylating STAT3 at Tyr705.

### 3.4 EEF2K facilitates melanoma progression by targeting SPP1

**FIGURE 4:**
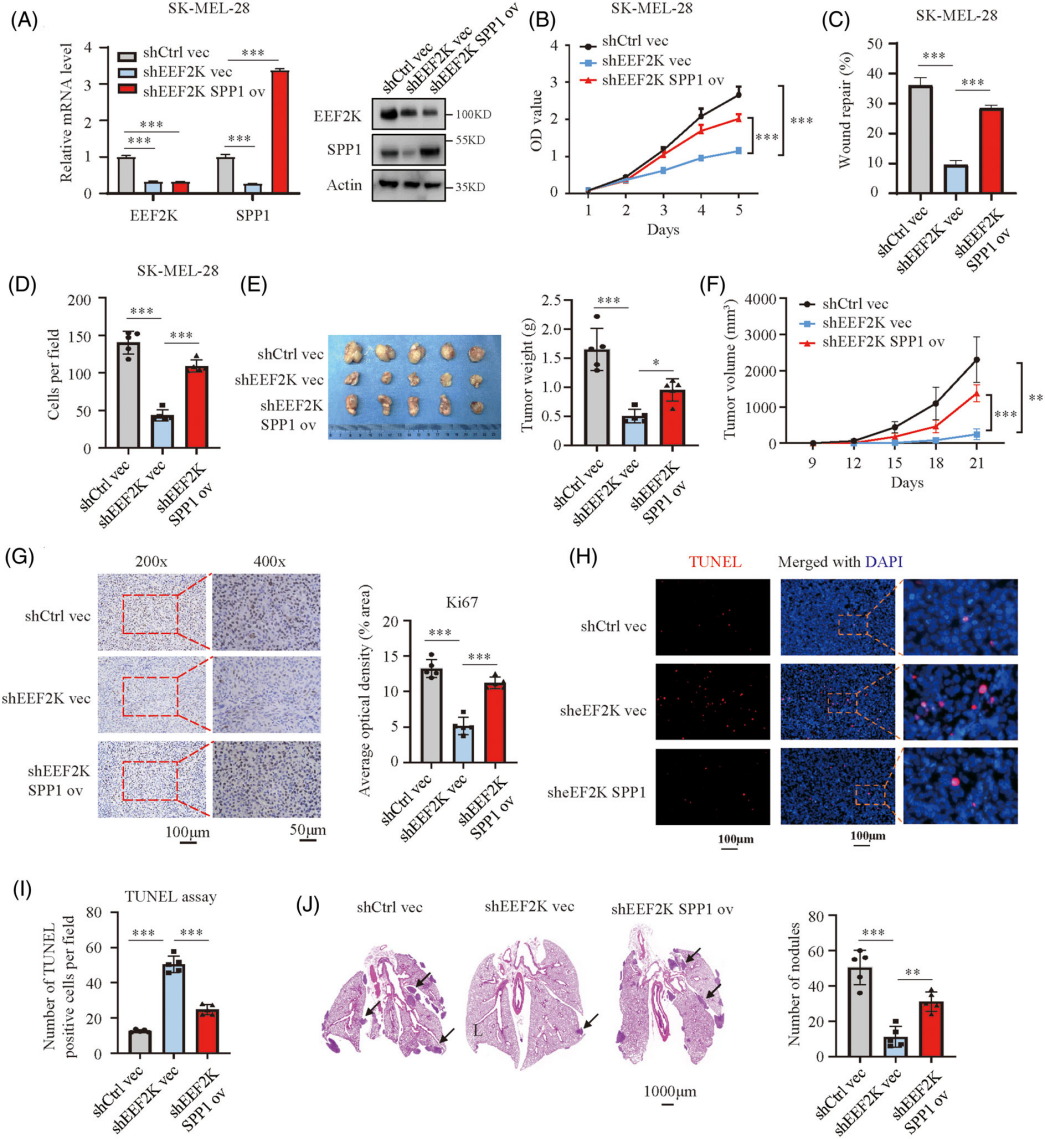
EEF2K facilitates melanoma progression by targeting SPP1. (A) Real‐time PCR and Western blotting analysis of EEF2K and SPP1 in the indicated SK‐MEL‐28 cells. (B–D) Cell proliferative capacity measured by cell counting kit‐8 assay (B), cell migratory capacity quantified by wound healing assay (C) and cell invasive capacity identified by transwell assay (D) of the indicated SK‐MEL‐28 cells. (E and F) Tumour weight (E) and tumour volume (F) in the indicated groups. (G) Ki67 staining of the sectioned tumours to identify tumour cell proliferation in the indicated groups. (H and I) TUNEL assay to quantify apoptotic cells in xenografted tumours. (J) Metastatic nodules in lung indicated by haematoxylin–eosin staining. *p*‐Values were determined using one‐way ANOVA analysis in (A), (C–E), (G), (I) and (J). Two‐way ANOVA was performed in (B) and (F). *p < .05; **p < .01; ***p < .001

Based on these results, we speculated that SPP1 functions as an effector of EEF2K in the context of melanoma progression. To test this hypothesis, we overexpressed SPP1 in EEF2K‐silenced SK‐MEL‐28 cells. The inhibition of SPP1 caused by EEF2K silencing was abrogated at both the mRNA and protein levels after SPP1 overexpression (Figure 4A). SPP1 overexpression rescued cell proliferative, migratory and invasive capabilities in EEF2K‐silenced SK‐MEL‐28 cells (Figure 4B–D). This finding was further validated using SPP1 silencing in EEF2K‐overexpressing A375 cells (Figure S5A). The enhanced proliferative, migratory and invasive capacities after EEF2K overexpression were notably abrogated by SPP1 depletion (Figure S5B–D). These results demonstrated that the inhibition of melanoma progression by EEF2K silencing is at least partially dependent on SPP1.

We further investigated whether these findings are reproducible in vivo. NSG mice were subcutaneously injected with SK‐MEL‐28 cells expressing shcontrol + vector, shEEF2K + vector or shEEF2K + SPP1. By comparing to the control group, tumours in the shEEF2K + vector group developed much slower, but this effect was abrogated by SPP1 overexpression (shEEF2K + SPP1 group) (Figure 4E,F and Figure S5E). Moreover, Ki67 IHC staining and TUNEL assay for the excised tumours indicated a significant increase in cell proliferation and decrease in apoptosis when SPP1 was overexpressed in EEF2K‐deficient melanoma cells (Figure 4G–I). In the lung metastasis model, EEF2K silencing decreased the metastatic burden and maintained a normal lung appearance, and the protective effects of EEF2K silencing were negated significantly by SPP1 overexpression (Figure 4J). Taken together, these results supported the notion that SPP1 contributes to EEF2K‐mediated melanoma progression.

### 3.5 EEF2K regulates SPP1 in a STAT3‐dependent manner

**FIGURE 5:**
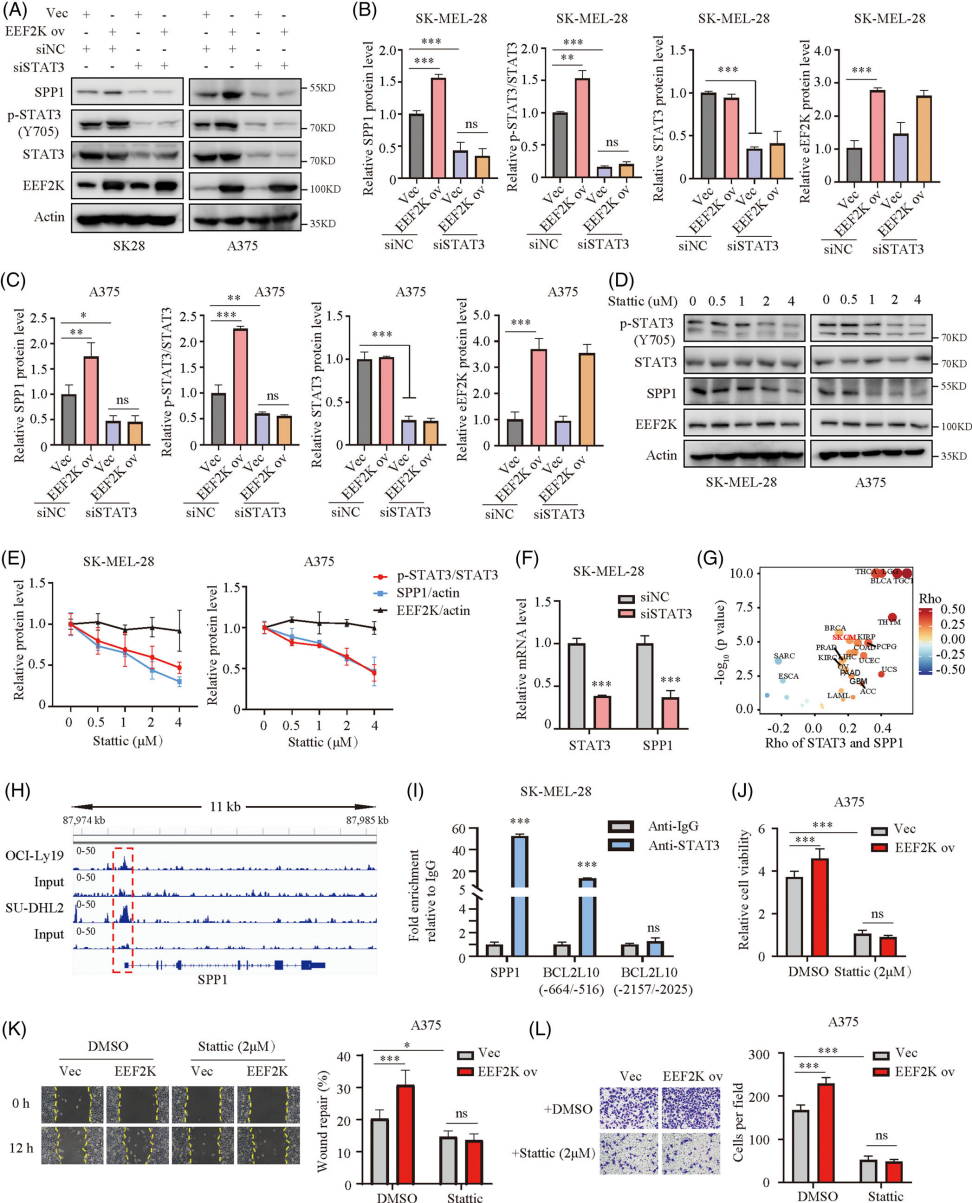
EEF2K regulates SPP1 in a STAT3‐depedent manner. (A–C) The protein levels of SPP1, p‐STAT3 and STAT3 in stably transfected SK‐MEL‐28 or A375 cells transduced with siNC or siSTAT3 were determined by Western blotting. (D and E) The protein levels of p‐STAT3, STAT3, SPP1 and EEF2K in SK‐MEL‐28 and A375 cells treated with stattic (STAT3 inhibitor) for 36 h quantified by Western blotting. (F) Real‐time PCR analysis of STAT3 and SPP1 in SK‐MEL‐28 cells after STAT3 silencing with siRNA. (G) Correlation analysis of STAT3 and SPP1 from the The Cancer Genome Atlas database. (H) Genome browser view of binding peaks on the SPP1 promoter in STAT3 ChIP and input control of OCI‐Ly19 cells and SU‐DHL2 cells. (I) ChIP‐qPCR analysis of the SPP1 promoter in SK‐MEL‐28 cells with STAT3 antibody or IgG. Primer of BCL2L10 (−664/−516) was used as a positive control and BCL2L10 (−2157/−2025) as a negative control. (J–L) Effect of stattic on role of EEF2K overexpression in A375 cell proliferation (48 h after 2 μM stattic exposure) (J), migration (12 h after 2 μM stattic exposure) (K) and invasion (18 h after 2 μM stattic exposure) (L). Two‐way ANOVA analysis was performed in (B), (C) and (J–L). Two‐tailed unpaired Student's *t*‐test was performed in (F) and (I). ns, no significance. *p < .05; **p < .01; ***p < .001

EEF2K is well known as a regulator of protein synthesis through inactivating EEF2 to control tumour initiation and growth.^21^, ^22^ To uncover the mechanism of EEF2K regulating SPP1, we knockdown EEF2 in both A375 and SK‐MEL‐28 cells (Figure S6). Unexpectedly, SPP1 expression remained unchanged after EEF2 silencing (Figure S6), indicating that SPP1 regulation by EEF2K is in an EEF2‐independent manner. Given that EEF2K positively regulates both SPP1 and p‐STAT3 (Figure 3 and Figure S4) and SPP1 is positively associated with p‐STAT3 in melanoma,^23^ we sought to verify whether EEF2K regulates SPP1 in a STAT3‐dependent manner. To test this hypothesis, we first examined the effect of STAT3 on SPP1 using an immunoblotting method. After silencing STAT3 with siRNA, as indicated by a decrease in STAT3 and p‐STAT3, SPP1 was significantly decreased even in EEF2K‐overexpressing melanoma cells (Figure 5A–C). Stattic, an inhibitor of STAT3, known for impeding the phosphorylation of STAT3 at Tyr705, profoundly attenuated the expression of SPP1 without influencing the expression of EEF2K (Figure 5D,E). These results suggested that EEF2K regulates SPP1 expression in a STAT3‐dependent manner.

To clarify the underlying mechanism by which STAT3 regulates SPP1 expression, we assessed the mRNA level of SPP1 after STAT3 silencing. Results showed that silencing of STAT3 with siRNA dramatically decreased SPP1 mRNA level (Figure 5F). Consistently, based on TCGA datasets, STAT3 was positively correlated with SPP1 in multiple types of cancer, including melanoma (Figure 5G). Analysis of published ChIP‐seq data showed that there was a strikingly enhanced STAT3‐binding peak on the SPP1 promoter of OCI‐Ly19 and SU‐DHL2 cells (Figure 5H). Based on the identified peak, we designed primers for ChIP‐qPCR analysis, aiming to validate that STAT3 binds to the SPP1 promoter in SK‐MEL‐28 melanoma cells (Figure 5I). Immunoprecipitation of STAT3 enabled specific amplification of BCL2L10 DNA fragments corresponding to regions −664 to −516, which served as a positive control, but not of the fragment −2157 to −2025, which served as a negative control (Figure 5I).^24^ These findings indicated that STAT3 regulates SPP1 expression at the transcriptional level.

To explore whether STAT3 contributes to EEF2K‐mediated melanoma progression, we further evaluated the effect of EEF2K on melanoma cells in the presence of stattic. The increased proliferative, migratory and invasive capacities induced by EEF2K overexpression were profoundly abolished by stattic (Figure 5J–L). Taken together, these results suggested that EEF2K promotes melanoma progression through the STAT3/SPP1 pathway.

### 3.6 Inhibition of EEF2K synergises with BET inhibitors in melanoma cells

**FIGURE 6:**
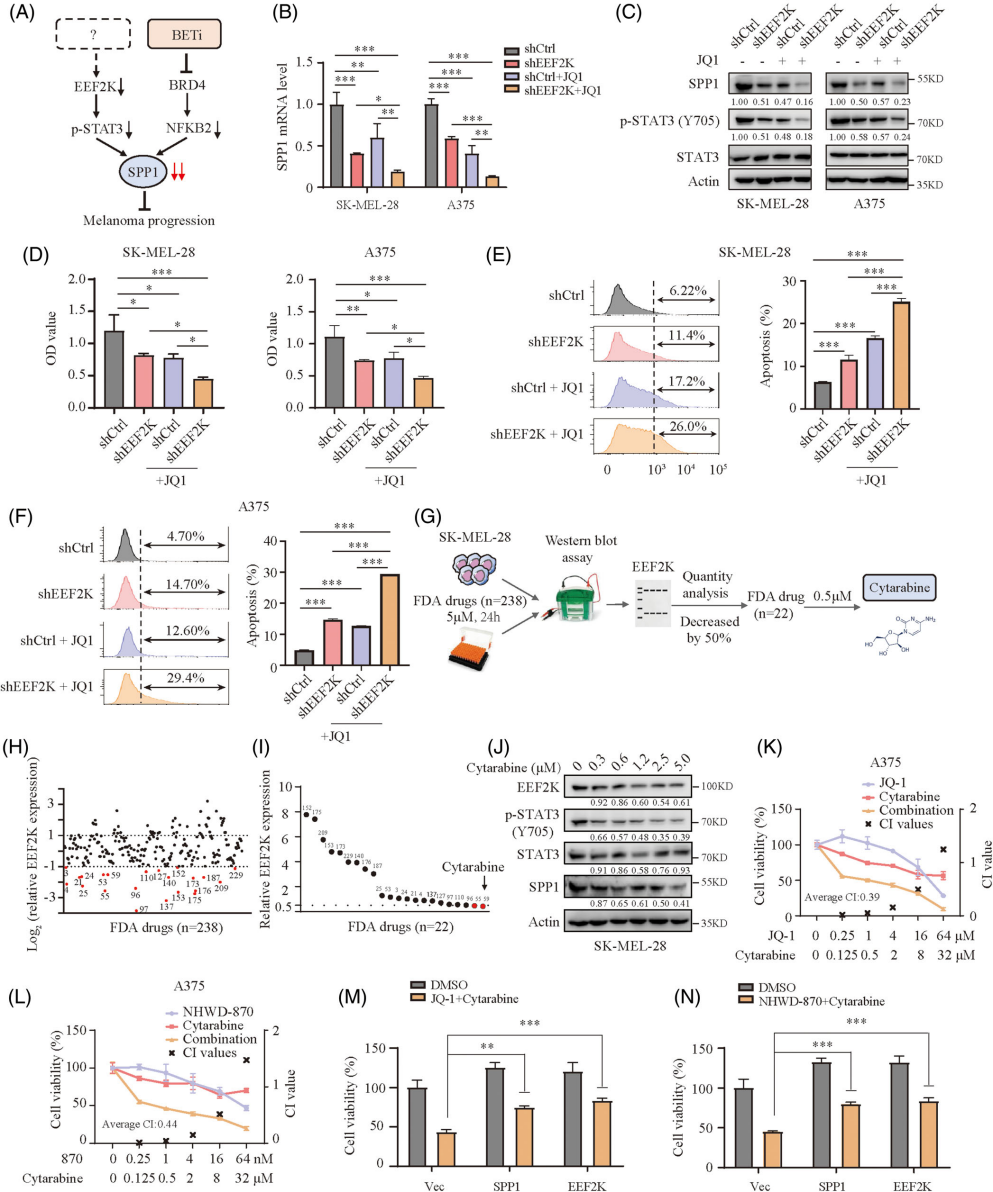
Inhibition of EEF2K synergises with BET inhibitors in melanoma cells. (A) A proposed model for the potential combination therapy of EEF2K inhibitor and BET inhibitor. (B) Real‐time PCR analysis of SPP1 mRNA level in SK‐MEL‐28 or A375 cells transfected with shCtrl or shEEF2K and then exposed to 1 μM JQ‐1 for 36 h. (C) The protein levels of SPP1, p‐STAT3 and STAT3 in SK‐MEL‐28 or A375 cells transfected with shCtrl or shEEF2K and then exposed to 1 μM JQ‐1 for 36 h. (D–F) Cell proliferation (48 h after JQ‐1 exposure) (D) and cell apoptosis (36 h after JQ‐1 exposure) (E and F) in SK‐MEL‐28 or A375 cells transfected with shCtrl or shEEF2K and then exposed to 1 μM JQ‐1. (G) A flowchart of Western blotting‐based drug screening. (H) A first screening with concentration of 5 μM for 24 h identified 22 out of 238 compounds that decreased EEF2K expression by 50%. (I) The second screening with concentration of 0.5 μM for 24 h identified cytarabine as the most efficient EEF2K inhibitor. (J) The protein levels of EEF2K, p‐STAT3, STAT3 and SPP1 in SK‐MEL‐28 cells treated with cytarabine for 24 h quantified by Western blotting. (K and L) Dose–response curves of A375 cells treated with cytarabine or JQ1 (K)/NHWD‐870 (L) either alone or in combination for 36 h (JQ1 and cytarabine at a fixed ratio of 2:1, NHWD‐870 and cytarabine at a fixed ratio of 1:500). (M and N) Cell viability of A375 cells transfected with vector, SPP1 or EEF2K plasmid and then exposed to 1 μM JQ‐1 (M)/10 nM NHWD‐870 (N) and 0.5 μM cytarabine for 36 h. CI, combination index. One‐way ANOVA analysis was performed in (B). Two‐way ANOVA analysis was performed in (D–F), (M) and (N). *p < .05; **p < .01; ***p < .001

We previously reported that BET inhibitors repressed melanoma progression through the nuclear factor kappa B subunit 2 (NFKB2)/SPP1 pathway.^15^ Now, we have validated that EEF2K silencing inhibits melanoma progression by repressing the STAT3‐SPP1 axis (Figure 6A). We further verified if BET inhibitors were more effective in suppressing SPP1 expression in EEF2K‐silenced cells. As expected, JQ‐1 treatment combined with EEF2K silencing significantly inhibited SPP1 expression, compared to a single treatment of EEF2K knockdown or BET inhibitor (Figure 6B,C). Furthermore, BET inhibitor treatment combined with EEF2K silencing significantly suppressed melanoma cell proliferation (Figure 6D and Figure S7A). In line with these findings, the percentage of apoptotic cells and cell arrest in the G0/G1 phase were markedly increased in EEF2K‐silenced melanoma cells treated with BET inhibitors (Figure 6E,F and Figure S7B–E). These results showed that EEF2K inhibition enhanced the suppressive effects of BET inhibitors on melanoma cells.

In terms of clinical applicability, we wondered whether EEF2K inhibitors could enhance the suppressive effect of BET inhibitors. Evidently, EEF2K inhibitors are currently not clinically applicable due to their lack of specificity and efficiency.^25^ Nevertheless, to identify potential clinically applicable EEF2K inhibitor candidates, we screened the 238 antitumour drugs identified from the US FDA‐approved drug library using immunoblotting assay for SK‐MEL‐28 cells (Figure 6G, Table S3). An initial screening using a higher concentration (5 μM) identified 22 compounds that could decrease EEF2K expression by 50% (Figure 6H and Figure S8). Subsequently, we performed a second screening using lower concentration (0.5 μM) and two compounds (cytarabine and crizotinib) that could reduce EEF2K expression by 50% were identified (Figure 6I and Figure S9A). Given that cytarabine could decrease EEF2K more effectively, it was chosen for our subsequent investigation. To further validate our screening results, cytarabine at different dosages was used to treat SK‐MEL‐28 melanoma cells, and the results showed that cytarabine repressed EEF2K, p‐STAT3 and SPP1 in a dose‐dependent manner (Figure 6J). As expected, the SPP1 expression was further decreased in melanoma cells after JQ‐1/NHWD‐870 and cytarabine treatment, compared with cytarabine or BET inhibitor treatment alone (Figure S9B). We subsequently treated SK‐MEL‐28 and A375 melanoma cells with various concentrations of BET inhibitors, cytarabine or BET inhibitors in combination with cytarabine for 36 h. Results demonstrated that BET inhibitors combined with cytarabine significantly reduced melanoma cell viability compared to single treatment with cytarabine or BET inhibitor (Figure 6K,L and Figure S9C,D). To confirm the synergistic effectiveness of the BET inhibitors and cytarabine combination, the combination index was estimated using the CompuSyn software. It was revealed that the combination indexes for the indicated concentrations of BET inhibitors and cytarabine were less than 1 in both A375 and SK‐MEL‐28 melanoma cells (Figure 6K,L and Figure S9C,D), suggesting the beneficial synergistic effect of BET inhibitors and cytarabine in the treatment of melanoma. Moreover, BET inhibitors combined with cytarabine significantly promoted apoptosis, as compared to a single treatment using cytarabine or BET inhibitor (Figure S9E–H). To interpret the role of EEF2K and SPP1 in mediating the synergy, we re‐examined the growth of A375 cells exposed to JQ‐1/NHWD‐870 and cytarabine after EEF2K or SPP1 overexpression, indicating that overexpression of either EEF2K or SPP1 can partially impede the synergy (Figure 6M,N).

### 3.7 Combination of cytarabine and BET inhibitors enhances the antitumour effect in vivo

**FIGURE 7:**
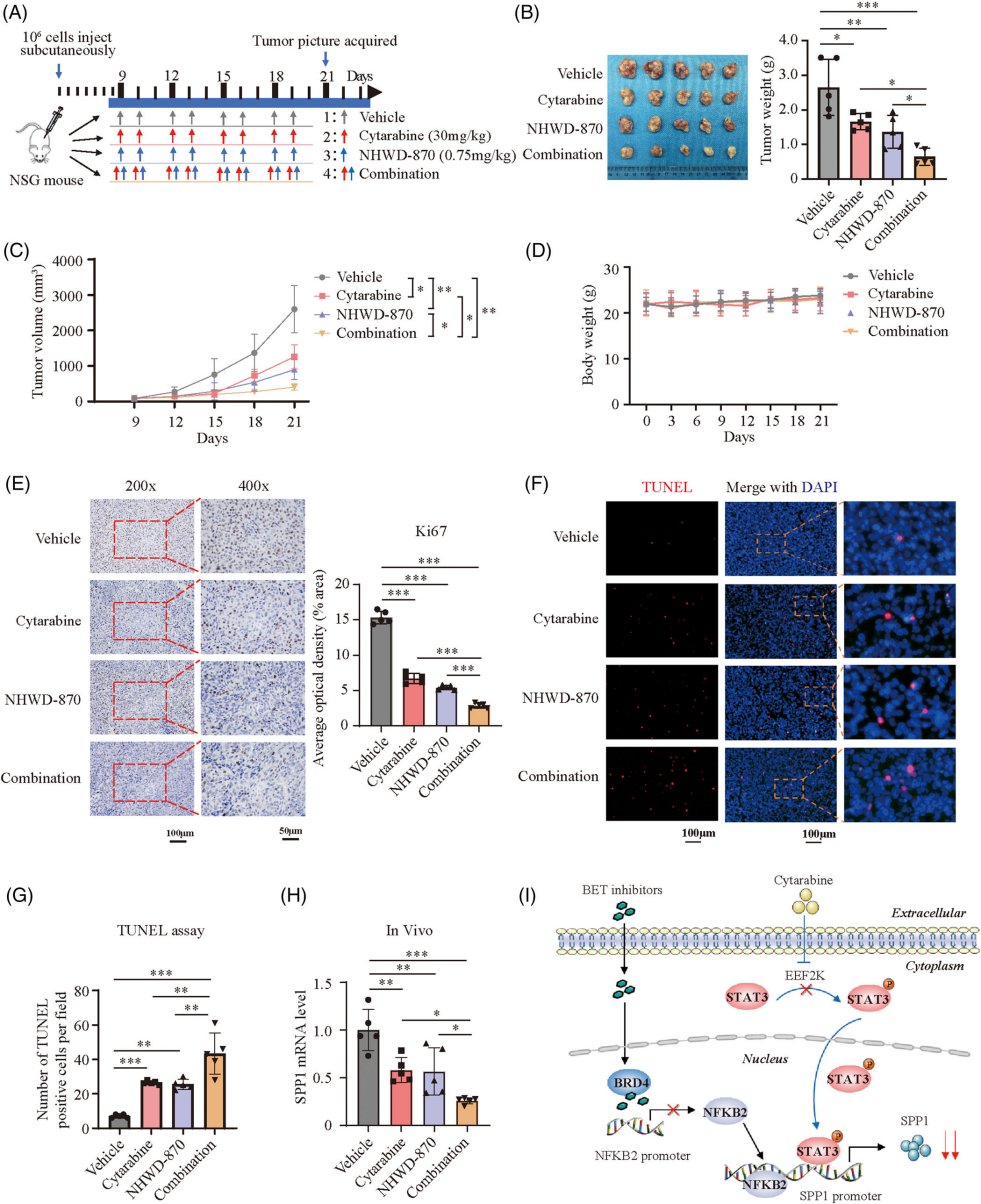
Combination of cytarabine and BET inhibitors enhance the antitumour effect in vivo. (A) Treatment schedule of tumour‐bearing mice for administration of cytarabine (30 mg/kg) and NHWD‐870 (0.75 mg/kg). (B–D) Tumour weight (B), tumour volume (C) and bodyweight (D) in control and treatment groups. (E) Ki67 staining of the sectioned tumours to identify tumour cell proliferation in control and treatment groups. (F and G) TUNEL assay to quantify apoptotic cells in xenografted tumours in control and treatment groups. (H) SPP1 mRNA level quantified by real‐time PCR in xenografted tumours. (I) A proposed working model. We previously identified that BET inhibitors inhibit SPP1 expression through BRD4/NFKB2 pathway (black line). In the present study, we found cytarabine can suppress EEF2K expression efficiently (blue line), resulting in further reduction of SPP1 expression when combined with BET inhibitor, thus enhancing the anticancer effect. *p*‐Values were calculated using one‐way ANOVA analysis in (B), (E) and (H) and using two‐way ANOVA analysis in (C). Brown–Forsythe and Welch ANOVA tests was used in (G). *p < .05; **p < .01; ***p < .001

To further evaluate the in vivo efficacy of combination therapy using cytarabine and BET inhibitors, SK‐MEL‐28 cells were inoculated into the right flank of NSG mice to generate subcutaneous xenograft models. When the tumour size reached 50–100 mm^3^, mice were randomly divided into the following four groups: vehicle (intraperitoneal injection of saline and corn oil administered via oral gavage), intraperitoneal injection of cytarabine (30 mg/kg), NHWD‐870 administered via oral gavage (0.75 mg/kg) or combination treatment using cytarabine and NHWD‐870. All treatments were administered for 2 successive days and stopped for 1 day (Figure 7A). Our data demonstrated that a single treatment using cytarabine or NHWD‐870 could reduce the rate of tumour growth, while the tumour volume and weight were further markedly decreased in the combination‐therapy group, without significant changes in bodyweight (Figure 7B–D). Further analysis on the isolated tumour tissue revealed markedly decreased proliferation and increased apoptosis in the combination‐therapy group, as validated by using Ki67 staining and TUNEL assay (Figure 7E–G). Real‐time PCR analysis of SPP1 in tumours showed that SPP1 expression was significantly decreased in the combination‐therapy group compared to either single‐treatment groups (Figure 7H). Taken together, our results strongly demonstrated that the combination therapy using cytarabine and BET inhibitors has immense therapeutic potential for melanoma treatment (Figure 7I).

## 4 DISCUSSION

The role of EEF2K in cancer and melanoma remains both controversial and obscure. To a certain extent, our results elucidated the oncogenic role of EEF2K in the growth and metastasis of melanoma. Specifically, in the combined therapy, EEF2K inhibitors exerted a synergistic effect on BET inhibitors that target SPP1. As a calcium/calmodulin‐dependent protein kinase, EEF2K is widely expressed in most tumours but moderately expressed in melanoma (Figure S10A). IHC staining revealed that EEF2K was differentially expressed in melanoma (Figure S10B). Analysis of Xiangya melanoma datasets based on previous reports^15^ suggested that overexpression of EEF2K is a prognostic factor for poor overall survival outcome (Figure S10C). Here, we identified that EEF2K/p‐STAT3/SPP1 may be a novel oncogenic pathway in melanoma progression and could be a potential target for a novel combinational therapy for melanoma.

Consistent with the results of previous studies,^26^, ^27^, ^28^ we found that EEF2K silencing promoted melanoma cell apoptosis and cell cycle arrest at G0/G1 phase. Moreover, we showed that disruption of EEF2K after BET inhibitor treatment largely attenuated the proliferation capacity of melanoma cells, which led to increased apoptosis and cell cycle arrest in the G0/G1 phase. Considering the current limitations of EEF2K inhibitors for clinical application, we screened the US FDA‐approved drug library for antitumour compounds and identified cytarabine as a potential clinically applicable EEF2K inhibitor that has a synergistic effect with BET inhibitors in melanoma treatment.

Several mechanisms have been proposed for the cytoprotective role of EEF2K in tumour development. In addition to the classic role of EEF2K in eukaryotic resource conservation by regulating protein synthesis, EEF2K may facilitate the synthesis of key proteins and thus promote cell survival under resource‐limited circumstances such as hypoxia and nutrient deprivation. Additionally, EEF2K could increase the supply of amino acids by inducing autophagy.^4^ Collectively, these mechanisms account for the cytoprotective role of EEF2K in tumour development. However, an opposite role of EEF2K in colorectal cancer has been reported, in which EEF2K could be directly inhibiting key protein synthesis or indirectly suppressing the activation of autophagy via accumulated cellular ATP levels.^10^ In our study, we showed that EEF2K functions as a tumour driver independent of its role in protein synthesis or autophagy induction.

Constitutive activation of STAT3 has been demonstrated to be an oncogenic feature in numerous tumours, including melanoma.^29^, ^30^, ^31^ The regulatory effect of EEF2K on STAT3 was contradictory in two previous studies. Zhou et al. reported that EEF2K silencing in HUVECs suppressed the level of p‐STAT3 in the culture medium containing liver cancer cells without clarifying the detailed mechanisms.^32^ In contrast, Xiao et al. indicated that EEF2K formed a complex with PKM2 and STAT3 to attenuate p‐STAT3 by phosphorylating PKM2 and inhibiting its dimerisation in lung cancer.^11^ Moreover, Cheng et al. previously demonstrated that EEF2K could inhibit the expression of PKM2 in breast cancer.^20^ However, comparable PKM2 expression in control versus EEF2K silencing cells and lack of interaction between EEF2K and PKM2 suggested that EEF2K increases the level of p‐STAT3 in a PKM2‐independent manner in melanoma. Intriguingly, in vitro kinase assay demonstrated that EEF2K, as a protein kinase, could directly phosphorylate STAT3 at Tyr705. These findings suggested that EEF2K plays different roles in different cell types. Furthermore, we found that p‐STAT3 could bind to the promoter region of SPP1 and enhance transcription, thus facilitating melanoma progression.

We previously identified SPP1 as a melanoma driver and found a strong association between SPP1 overexpression and poor prognosis. Other studies have demonstrated that SPP1 could facilitate melanoma cell invasion by activating integrin alphavbeta3 and downregulating tetraspanin CD9.^33^ In the tumour microenvironment, SPP1 could also activate macrophages, induce angiogenesis and promote melanoma growth.^34^ However, the upstream regulative role of SPP1 has not been fully investigated. Previously, Wu et al. demonstrated a strong positive association between p‐STAT3 and SPP1 in melanoma samples,^23^ but they did not clarify the regulatory mechanism. Goel et al. suggested that STAT3 could induce SPP1 expression but only at the distal enhancer region of SPP1 in hyper‐IgE syndrome.^35^ Moreover, increased SPP1 was found to upregulate p‐STAT3, which activated STAT3 signalling in osteosarcoma and rat hepatocytes.^36^, ^37^ Here, we demonstrated that EEF2K regulates SPP1 in a STAT3‐dependent manner and p‐STAT3 binds to the promoter region of SPP1 to enhance its transcription.

We previously identified that BET inhibitors could suppress melanoma progression by downregulating SPP1 expression. However, BET inhibitors alone exhibited limited efficacy against solid tumours, highlighting the significance of combination therapy.^38^ We validated that EEF2K silencing could enhance the inhibitory effects of BET inhibitors on melanoma, probably by further downregulating SPP1 expression. Based on the US FDA‐approved drug library, we have further identified cytarabine, a drug used in the treatment of several types of leukaemia, which could decrease EEF2K expression significantly. Cytarabine was reported to mediate apoptosis through Rel A (p65) dephosphorylation.^39^ Omsland and colleagues showed that cytarabine could also inhibit NF‐κB activity.^40^ Moreover, NF‐κB inhibition could decrease EEF2K expression in different cancer cell lines.^41^ These studies suggested that cytarabine might repress EEF2K expression through NF‐κB signalling pathway. A previous study reported that SPP1 neutralisation yielded a synergistic effect with cytarabine in the treatment of haematological malignancies.^42^ BET inhibitors have been reported to suppress SPP1 expression and here, we further validated the previous findings in melanoma. What is more, ARV‐825, degrader of BET proteins or JQ1 have been shown to synergise with cytarabine in inhibiting acute myeloid leukaemia. However, the mechanisms remain unclear.^43^, ^44^ Consistent with the previous findings, we showed that BET inhibitors could synergise with cytarabine in the treatment of melanoma. Mechanistically, we demonstrated that BET inhibitors and cytarabine exerts a synergetic effects through further repressing SPP1 expression, which filled a previously unidentified mechanism gap. BET inhibitors are currently in the preclinical stage of drug study, and our results further warranted its potential in clinical application.

In conclusion, our study demonstrated that EEF2K plays an oncogenic role in melanoma progression via the p‐STAT3/SPP1 axis. Moreover, EEF2K inhibition significantly reinforced the antitumour efficiency of BET inhibitors. Our findings not only elucidated a new molecular target for melanoma therapy but also highlighted a potential novel combination therapy strategy.

## CONFLICT OF INTEREST

The authors declare that there is no conflict of interest.

## Supporting information



Supporting information

Supporting information

Supporting information

Supporting information
