# Supplementary material for: EEF2K silencing inhibits tumour progression through repressing SPP1 and synergises with BET inhibitors in melanoma
Source: Clin Transl Med. 2022 Feb 20;12(2):e722. doi: 10.1002/ctm2.722 (PMC8858631; doi:10.1002/ctm2.722)
Supplement: Supplementary file 1 — Supporting information [file CTM2-12-e722-s001.docx]

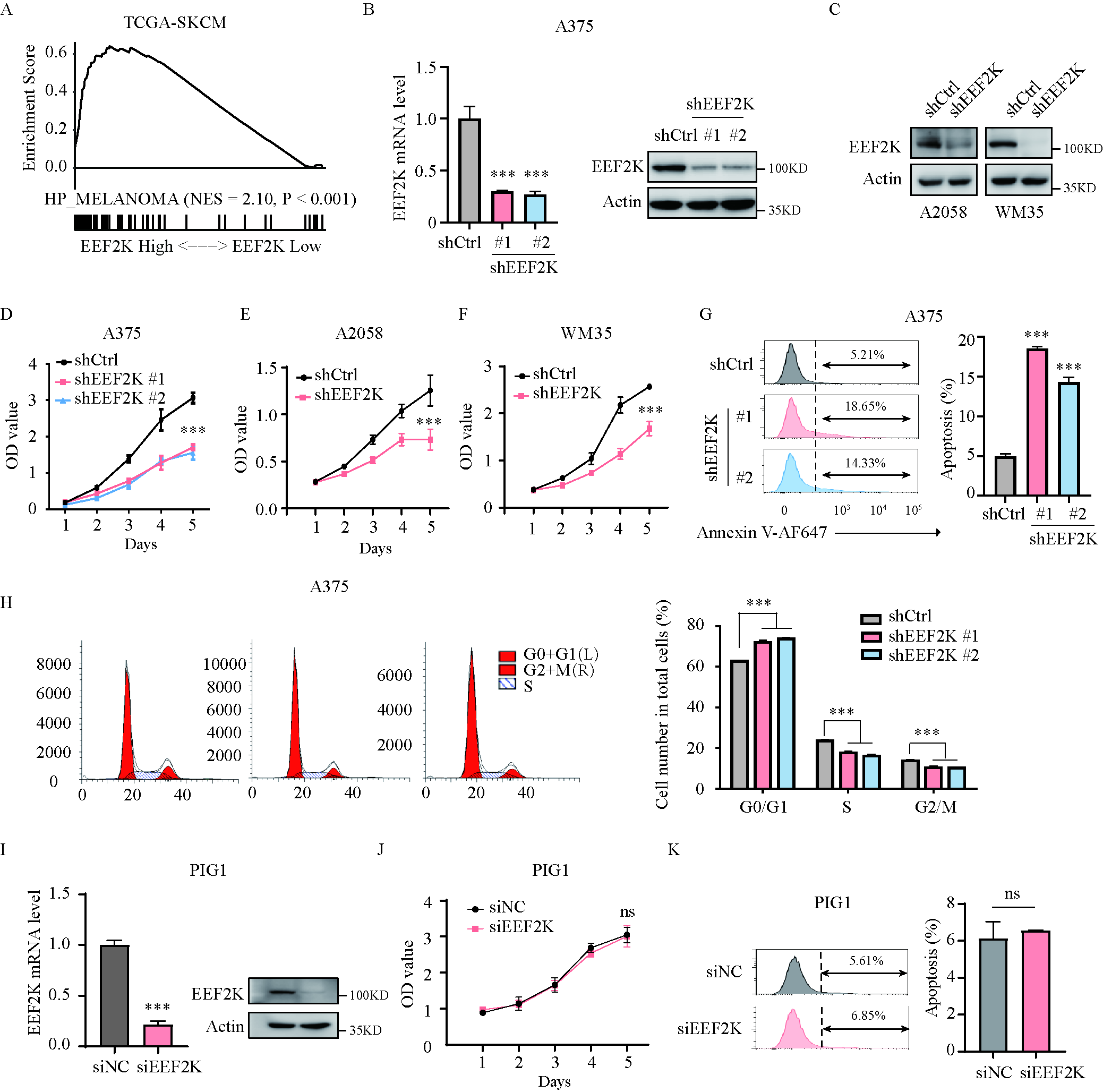


**Figure S1. EEF2K silencing blocks cell growth, causes cell apoptosis and cell cycle arrest.** (**A**) Gene set enrichment analysis according to EEF2K expression profile in TCGA-SKCM dataset. (**B**) EEFK2 knockdown efficiency in A375 cells quantified by real-time PCR and western blotting. (**C**) EEFK2 knockdown efficiency in A2058 and WM35 cells quantified by western blotting. (**D-F**) Cell proliferation of the indicated cells after EEF2K silencing. (**G-H**) Cell apoptosis (**G**), and cell cycle distribution (**H**) of A375 cells after EEF2K silencing. (**I**) EEFK2 knockdown efficiency in PIG1 cells quantified by real-time PCR and western blotting. (**J-K**) Cell proliferation (**J**) and cell apoptosis (**K**) of PIG1 cells after EEF2K silencing. One-way ANOVA analysis was performed in **B, G, H**. Two-way ANOVA analysis was performed in **D, E, F, J**. Two-tailed unpaired Student’s t-test was performed in **K**. Nonparametric test was performed in **I**. ns, no significance. ***, P < 0.001.


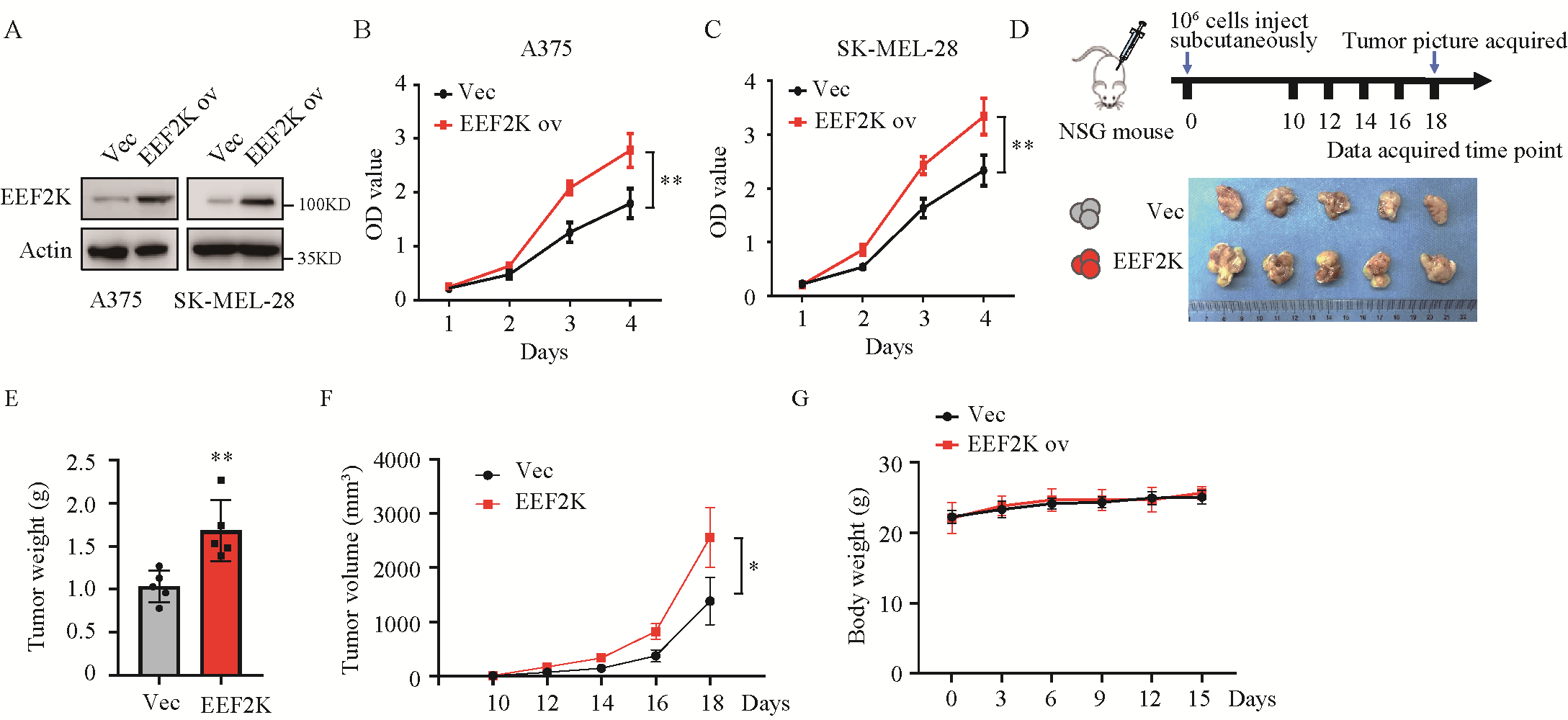


**Figure S2. EEF2K overexpression promotes cell growth in vitro and in vivo.** (**A**) EEF2K overexpression effciency quantified by western blotting in A375 and SK-MEL-28 cells. (**B-C**) Cell proliferation of A375 (**B**) and SK-MEL-28 (**C**) cells after EEF2K overexpression. (**D**) Schematic view of the xenografted model and picture of the resected xenografted tumors (n = 5 in each group). (**E-G**) Tumor weight (**E**), tumor volume (**F**), and body weight (**G**) of mice in the indicated groups. Two-way ANOVA analysis was performed in **B, C, F**. Two-tailed unpaired Student’s t-test was performed in **E**. *, P < 0.05; **, P < 0.01.


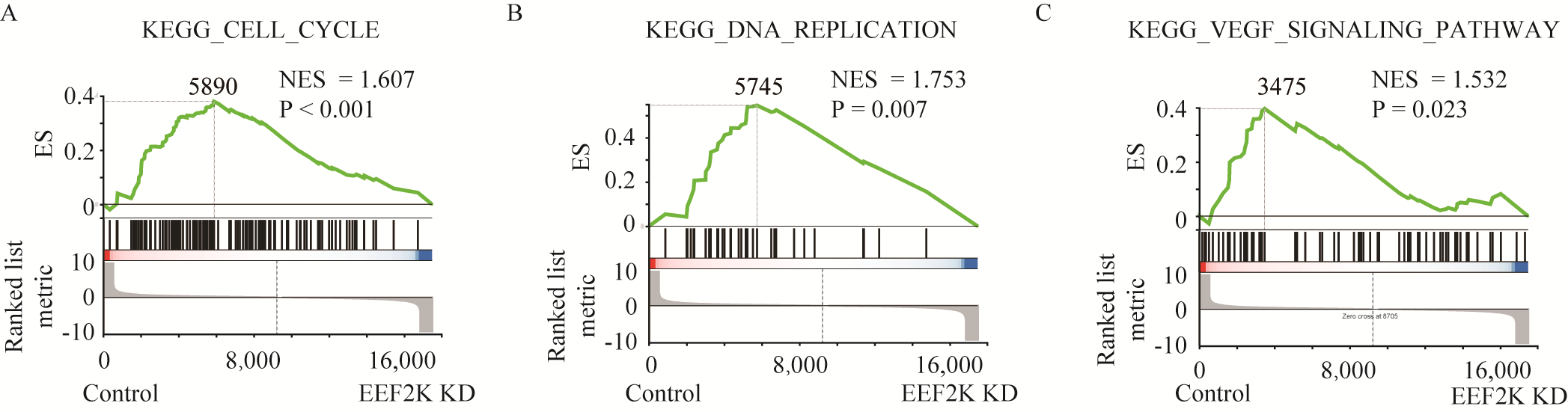


**Figure S3. Gene Set Enrichment Analysis in EEF2K knockdown compared to control SK-MEL-28 cells.** (**A-C**) Gene set enrichment analysis of cell cycle (**A**), DNA replication (**B**), and vascular endothelial growth factor signaling pathway (**C**).


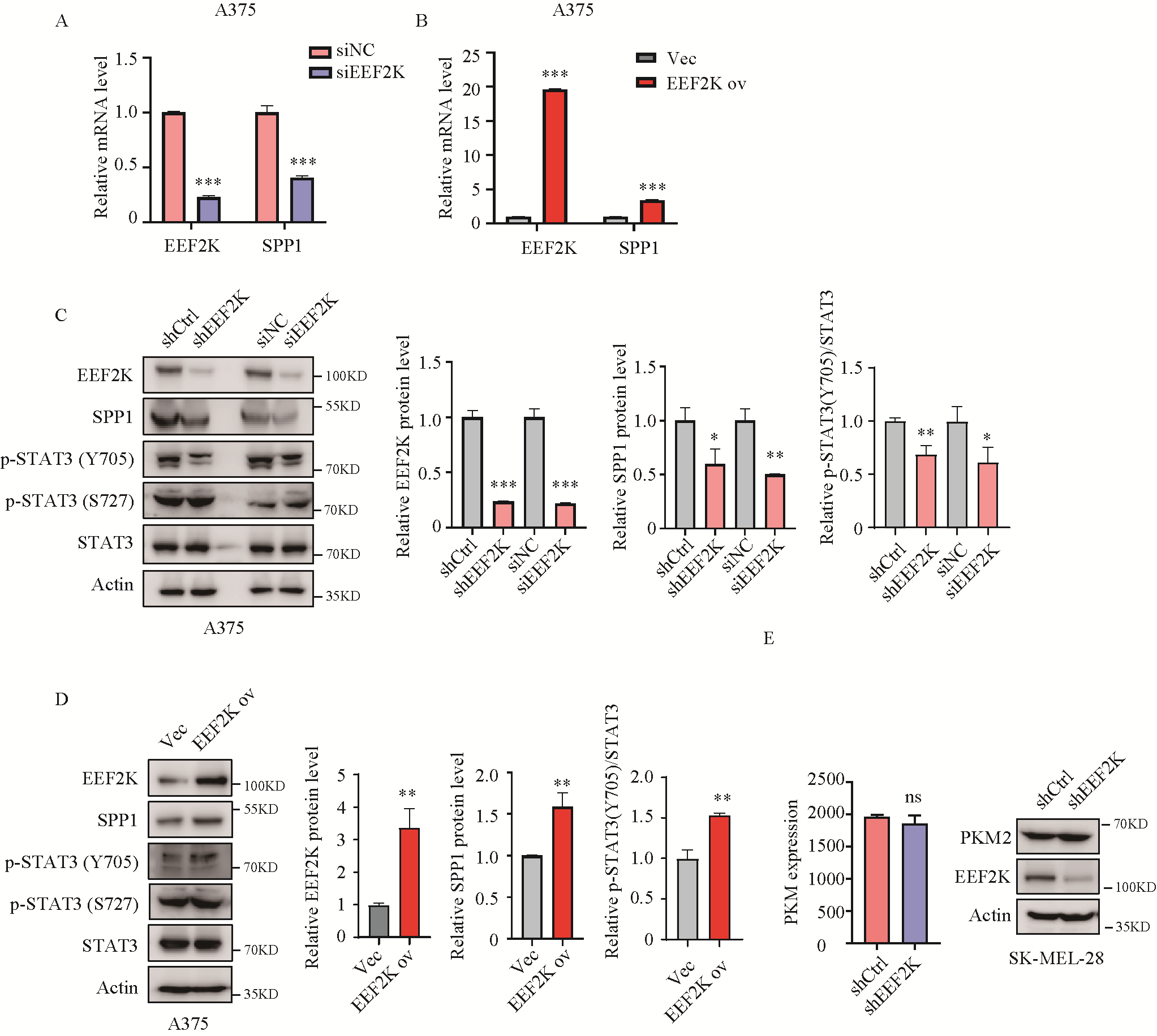


**Figure S4. EEF2K regulated SPP1 expression and STAT3 signaling pathway.** (**A-B**) SPP1 mRNA expression in A375 cells after EEF2K silencing with siRNA (**A**) or EEF2K overexpression (**B**). (**C**) Quantification by western blotting of SPP1, p-STAT3, and STAT3 expression after EEF2K knockdown in A375 cells. (**D**) Quantification by western blotting of SPP1, p-STAT3, and STAT3 expression after EEF2K overexpression in A375 cells. (**E**) The mRNA (from our RNA sequencing data) and protein level of PKM in SK-MEL-28 cells after EEF2K silencing. P values were calculated using two-tailed unpaired Student’s t-test or unpaired t test with Welch’s correction if the standard deviations were unequal. ns, no significance. *, P < 0.05; **, P < 0.01; ***, P < 0.001.


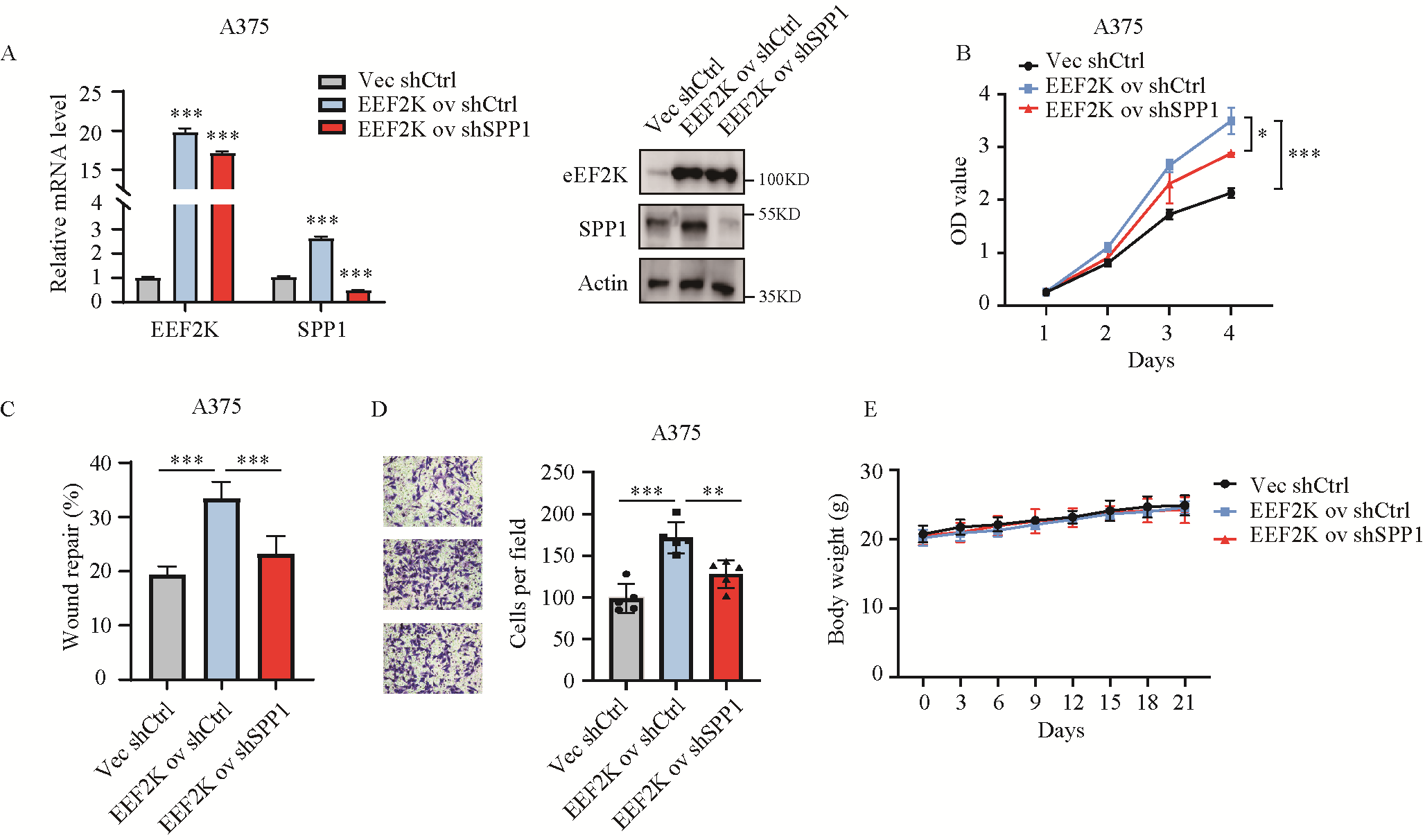


**Figure S5. EEF2K regulated melanoma progression in a SPP1-dependent manner.** (**A**) Real-time PCR and western blotting analysis of EEF2K and SPP1 in the indicated A375 cells. (**B-D**) Cell proliferative capacities measured by cell counting kit-8 assay (**B**), cell migratory capacities quantified by wound healing assay (**C**), and cell invasive capacities identified by Transwell assay (**D**) of the indicated A375 cells. (**E**) Body weight of the mice in the indicated group. One-way ANOVA analysis was performed in **A, C, D**. Two-way ANOVA analysis was performed in **B**. *, P < 0.05; **, P < 0.01; ***, P < 0.001.


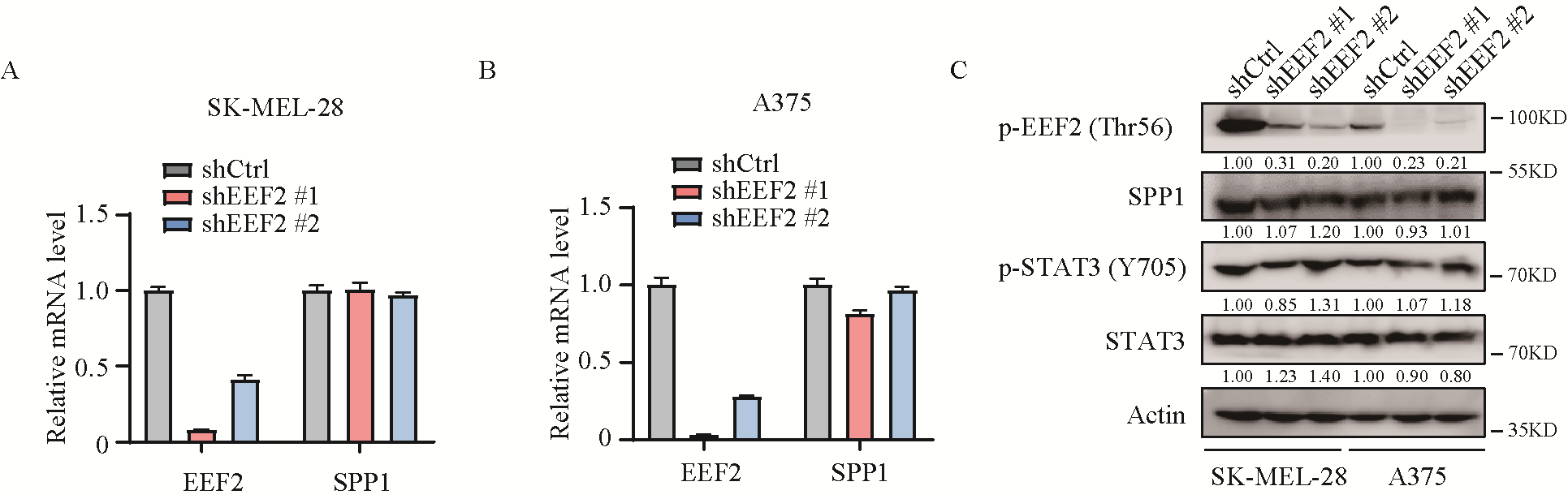


**Figure S6.** Regulation of SPP1 by EEF2K is independent of EEF2. (**A-B**) Evaluation by real-time PCR of SPP1 expression in SK-MEL-28 and A375 cells after EEF2 knockdown. (**C**) Evaluation by western blotting of SPP1 expression in SK-MEL-28 and A375 cells after EEF2 knockdown.


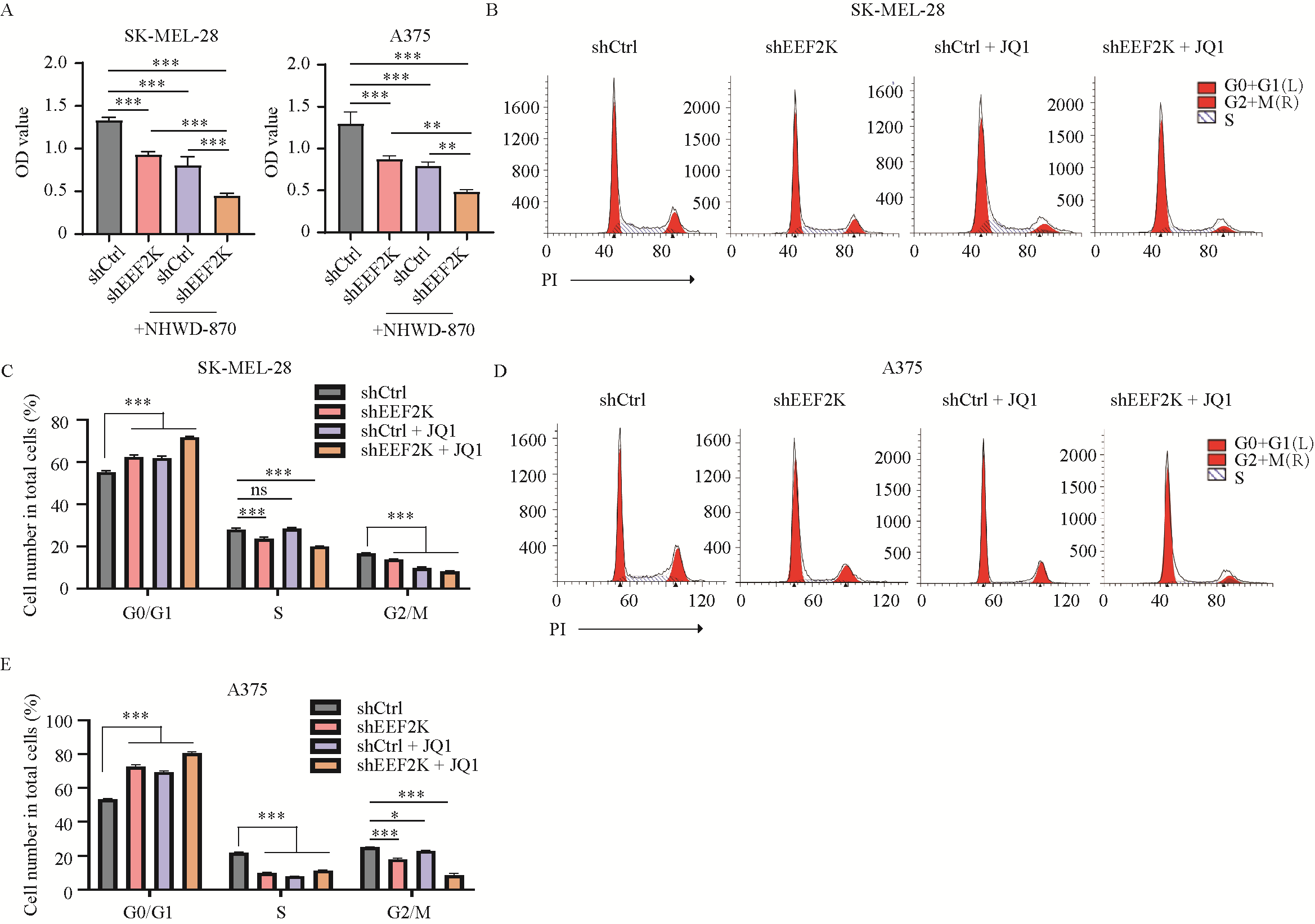


**Figure S7. Anticancer effect of EEF2K knockdown combined with BET inhibitors.** (**A**) Cell proliferation of SK-MEL-28 or A375 cells transfected with shCtrl or shEEF2K and then exposed to 10nM NHWD-870 for 48 h. (**B-E**) Cell cycle of SK-MEL-28 (**B-C**) or A375 (**D-E**) cells transfected with shCtrl or shEEF2K and then exposed to 1μM JQ-1 for 36 h. P values were calculated using two-way ANOVA analysis. ns, no significance. *, P < 0.05; **, P < 0.01; ***, P < 0.001.


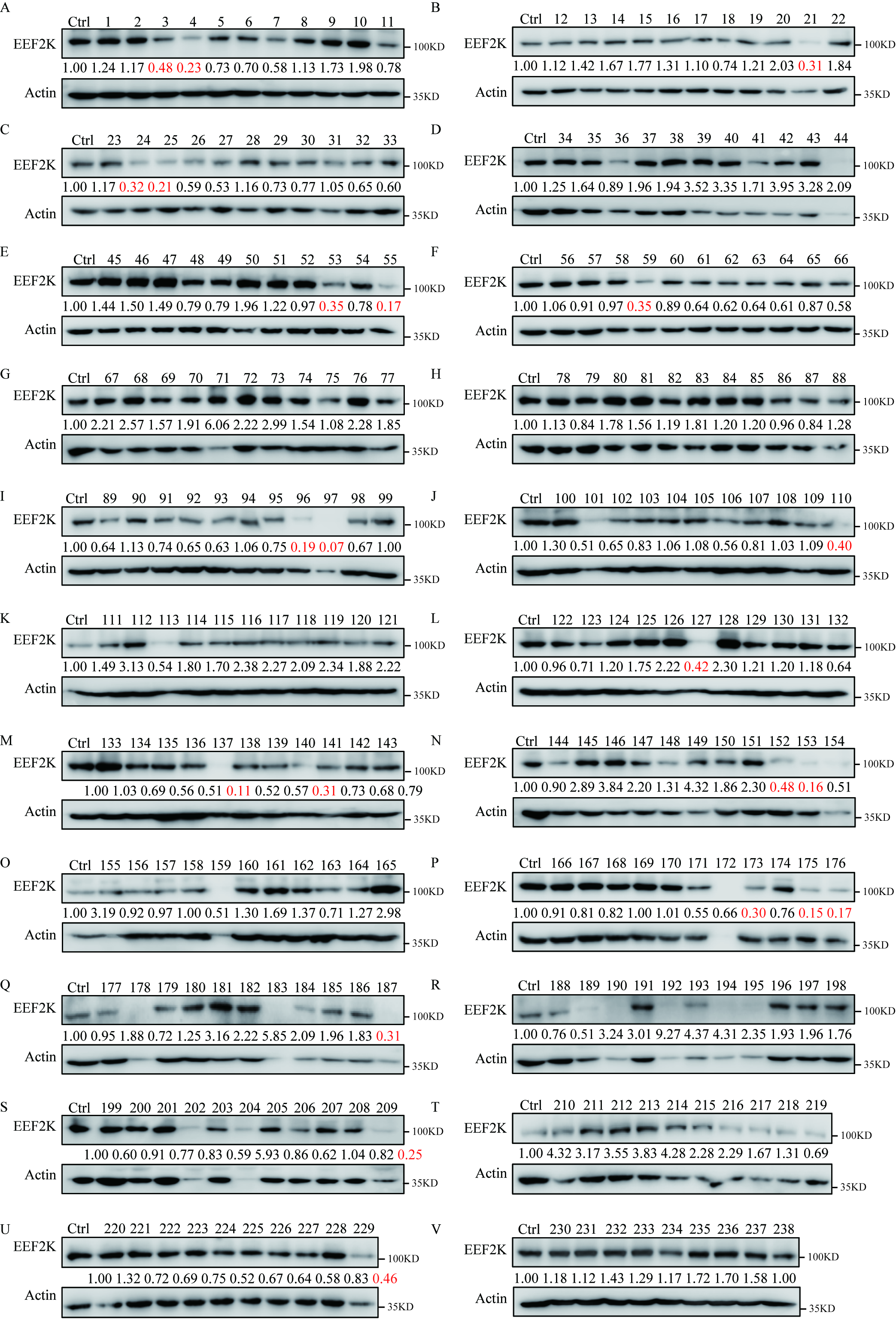


**Figure S8. First drug screening with concentration of 5μM for 24 h based on western blotting.**


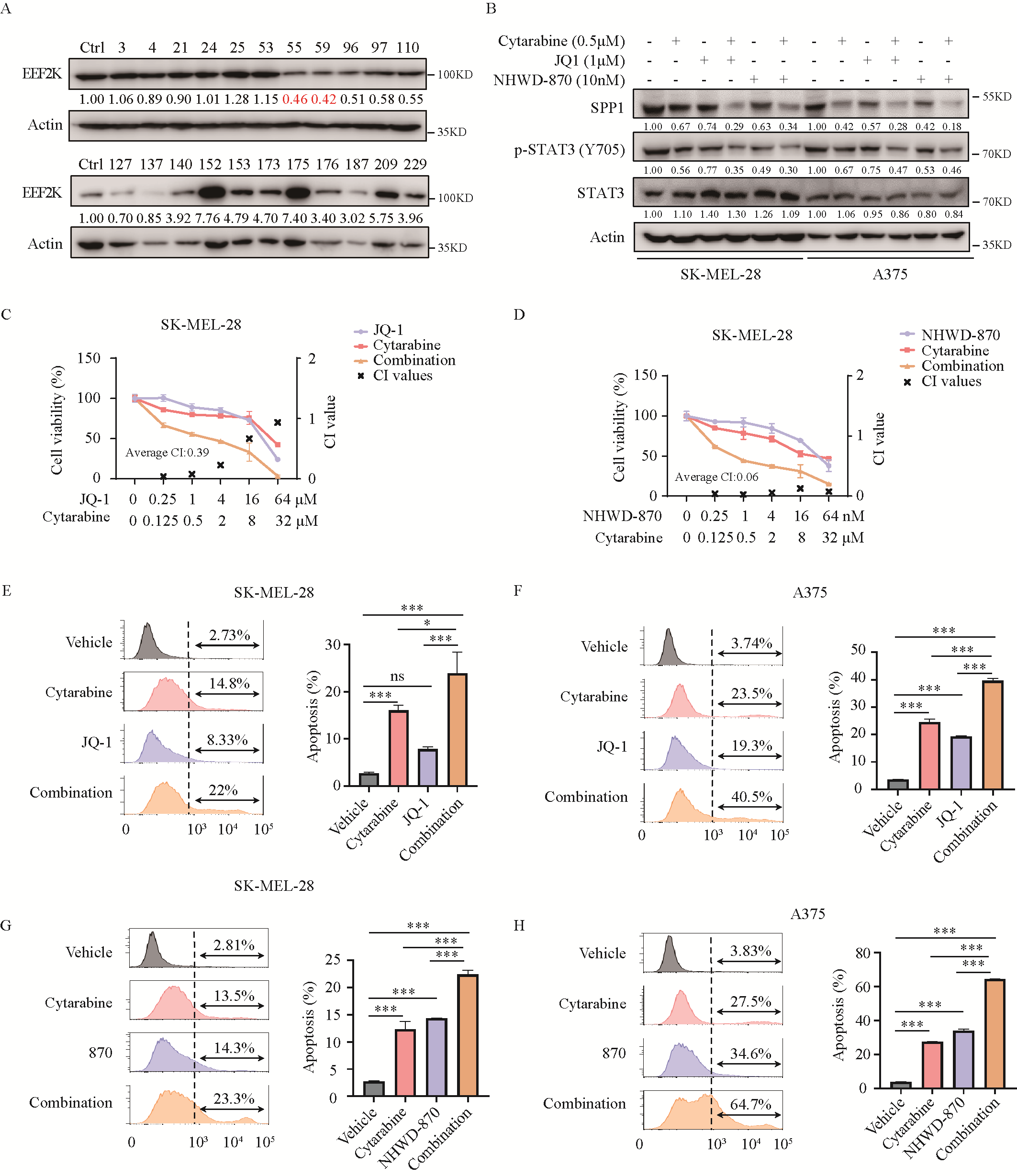


**Figure S9. Anticancer effect of cytarabine combined with BET inhibitors.** (**A**) Second drug screening with concentration of 0.5μM for 24 h based on western blotting. (**B**) Quantification by western blotting of SPP1 expression in melanoma cells after treatment for 36 h of 0.5μM cytarabine or 1μM JQ1/ 10nM NHWD-870 either alone or in combination. (**C-D**) Dose-response curves of SK-MEL-28 cells treated with cytarabine or JQ1 (**C**)/ NHWD-870 (**D**) either alone or in combination for 36 h ( JQ1 and cytarabine at a fixed ratio of 2:1, NHWD-870 and cytarabine at a fixed ratio of 1:500). (**E-H**) Cell apoptosis in SK-MEL-28 (**E, G**) or A375 (**F, H**) cells treated for 36 h with 0.5μM cytarabine or 1μM JQ1/ 10nM NHWD-870 either alone or in combination. P values were calculated using one-way ANOVA analysis. ns, no significance. *, P < 0.05; **, P < 0.01; ***, P < 0.001.


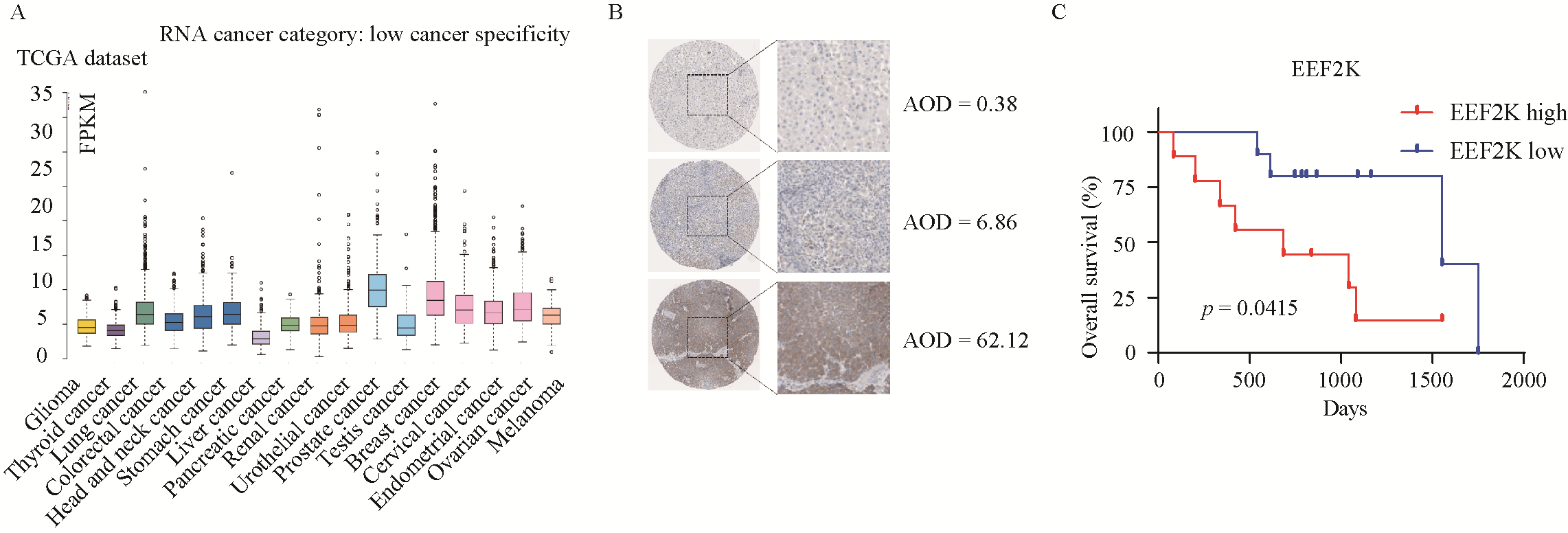


**Figure S10. EEF2K expression in melanoma and its prognostic value.** (**A**) Average EEF2K expression in various types of cancer from the Human Protein Atlas. (**B**) EEF2K expression quantified by immunohistochemistry from the Human Protein Atlas. AOD, average optical density. The expressions were quantified by Image J. (**C**) Kaplan-Meier survival analysis of high and low EEF2K expression groups in Xiangya melanoma cohort.
