# Supplementary material for: EEF2K silencing inhibits tumour progression through repressing SPP1 and synergises with BET inhibitors in melanoma
Source: Clin Transl Med. 2022 Feb 20;12(2):e722. doi: 10.1002/ctm2.722 (PMC8858631; doi:10.1002/ctm2.722)
Supplement: Supplementary file 2 — Supporting information [file CTM2-12-e722-s004.docx]

**Table S1. Sequence of primers used for real-time PCR.**

| Gene symbol | Forward primer | Reverse primer |
| --- | --- | --- |
| EEF2K | CCACACAGCCAGAAGCTAGA | ATGCCATGTTATCGAGGTCA |
| RNF11 | GCCTAAAGGAGTTTATGACCCTG | GTCCAGGTGATAGATGTGCATGC |
| ENPP2 | TATGCTGCGGAAACTCGTCAGG | GACGTTGACACACCGATGCAGT |
| BMP4 | CTGGTCTTGAGTATCCTGAGCG | TCACCTCGTTCTCAGGGATGCT |
| PDE2A | GCTGGTGAACAAGATCAATGGGC | GCTGCGATACTGAGCCTCATTC |
| PLCB2 | CCTGGAAGTGACGGCTTATGAG | GCTCTGTGAAGGACGAGATGAC |
| SPP1 | CGAGGTGATAGTGTGGTTTATGG | GCACCATTCAACTCCTCGCTTTC |
| BST2 | TCTCCTGCAACAAGAGCTGACC | TCTCTGCATCCAGGGAAGCCAT |
| ART3 | CCTTCAAAGCATAAACAAGACCTG | CAGGGTTGTAGACATAGATGGGT |
| NID1 | ACATTGAGCCCTACACGGAGCT | GCCACTGGTAAGTGTAGATGCG |
| NRCAM | TGTGGCTGAAGGACAACAGGGA | AGACGCTGTCCAGAGTGGTGTT |
| STAT3 | CTTTGAGACCGAGGTGTATCACC | GGTCAGCATGTTGTACCACAGG |
| GAPDH | AATCCCATCACCATCTTCCA | GTCATCATATTTGGCAGGTT |
