# Supplementary material for: EEF2K silencing inhibits tumour progression through repressing SPP1 and synergises with BET inhibitors in melanoma
Source: Clin Transl Med. 2022 Feb 20;12(2):e722. doi: 10.1002/ctm2.722 (PMC8858631; doi:10.1002/ctm2.722)
Supplement: Supplementary file 3 — Supporting information [file CTM2-12-e722-s002.docx]

**Table S2.** **Table summary of differentially expressed melanoma-promoting genes.**

| Gene symbol | Gene name | Log2FC | Q-value | PMID |
| --- | --- | --- | --- | --- |
| RNF11 | Ring finger protein 11 | -1.91327224 | 2.53898E-39 | 26550462 |
| ENPP2 | Ectonucleotide pyrophosphatase/phosphodiesterase 2 | -1.768869877 | 0.004592328 | 22986745 |
| BMP4 | Bone morphogenetic protein 4 | -1.630274021 | 0.040794833 | 17173062, 15695386, 20480203 |
| PDE2A | Phosphodiesterase 2A | -1.515134916 | 0.009814807 | 24705027 |
| PLCB2 | Phospholipase C beta 2 | -1.507142728 | 0.027604096 | 31746389 |
| SPP1 | Secreted phosphoprotein 1 | -1.474167608 | 1.40597E-10 | 33052224 |
| BST2 | Bone marrow stromal cell antigen 2 | -1.459464206 | 0.005310368 | 23702480 |
| ART3 | ADP-ribosyltransferase 3 (inactive) | -1.42759106 | 5.69067E-07 | 29725430 |
| NID1 | Nidogen 1 | -1.332386816 | 3.82632E-27 | 28827399 |
| NRCAM | Neuronal cell adhesion molecule | -1.251061099 | 9.38825E-11 | 16357171 |
| SDC4 | Syndecan 4 | -1.211289647 | 6.93999E-25 | 20803552 |
| UCP2 | Uncoupling protein 2 | -1.197654943 | 0.000609271 | 32319575 |
| HMGCS1 | 3-hydroxy-3-methylglutaryl-CoA synthase 1 | -1.151850839 | 1.39427E-07 | 28468827 |
| RAI14 | Retinoic acid induced 14 | -1.11590741 | 1.70802E-10 | 32228518 |
| BGN | Biglycan | -1.102539741 | 5.17478E-05 | 28476030 |
| UBE3C | Ubiquitin protein ligase E3C | -1.097366596 | 2.07001E-20 | 26894856 |
| TRIB2 | Tribbles pseudokinase 2 | -1.084869969 | 5.00457E-11 | 20208562, 25586991, 29670085 |
| CXCL1 | C-X-C motif chemokine ligand 1 | -1.083411367 | 7.02184E-08 | 20596077 |
| ADORA1 | Adenosine A1 receptor | -1.048969855 | 0.017389969 | 32183950 |
| DEPDC1B | DEP domain containing 1B | -1.044416747 | 1.03304E-08 | 30880030 |
| MYOF | Myoferlin | -1.001140129 | 1.71816E-15 | 29164766 |
| PAK1 | P21 (RAC1) activated kinase 1 | -1.000863782 | 5.43853E-13 | 29187213 |

FC, fold change.
